# Ginsenoside Rb1 ameliorates stress urinary incontinence by upregulating SIRT1 to preserve mitochondrial homeostasis and MAM integrity

**DOI:** 10.3389/fphar.2026.1912811

**Published:** 2026-10-02

**Authors:** Liying Chen, Lingyun Li, Mao Chen, Ya Xiao, Hanyue Li, Xiaoyu Tian, Li Hong, Jianming Tang

**Affiliations:** 1 Department of Gynecology and Obstetrics, Renmin Hospital of Wuhan University, Wuhan, Hubei Province, China; 2 Pelvic Floor Research Centre of Hubei Province, Renmin Hospital of Wuhan University, Wuhan, China

**Keywords:** ginsenoside Rb1, mitochondria-associated membranes, mitochondrial biogenesis, mitophagy, SIRT1, stress urinary incontinence

## Abstract

**Introduction:**

Stress urinary incontinence (SUI) is a prevalent pelvic floor disorder characterized by impaired connective tissue integrity. Oxidative stress-induced mitochondrial dysfunction has been implicated in pelvic floor degeneration. Ginsenoside Rb1 (Rb1), a bioactive component of ginseng, exhibits antioxidant properties; however, its therapeutic effects and underlying mechanisms in SUI remain unclear.

**Methods:**

A vaginal distension (VD)-induced mouse model of SUI and an H_2_O_2_-induced oxidative stress model in L929 cells were established to investigate the effects of Rb1. Urodynamic evaluation, histological analysis, mitochondrial functional assays, and SIRT1 inhibition/knockdown experiments were performed to explore the molecular mechanisms.

**Results:**

Rb1 significantly improved bladder leak point pressure, preserved collagen organization, and reduced apoptosis in the anterior vaginal wall of VD mice. Rb1 alleviated oxidative stress by reducing ROS accumulation and restoring mitochondrial homeostasis. Mechanistically, Rb1 upregulated SIRT1 and enhanced mitochondrial biogenesis-associated signaling through the PGC-1α/NRF1/TFAM pathway, while promoting PINK1/Parkin-associated mitophagic signaling. Furthermore, Rb1 maintained mitochondria-associated membrane (MAM) integrity by regulating GRP75 acetylation, thereby reducing excessive ER–mitochondrial calcium transfer, mitochondrial calcium overload, and mtROS accumulation. SIRT1 inhibition or knockdown attenuated these protective effects.

**Conclusion:**

Ginsenoside Rb1 protects against SUI by promoting SIRT1-associated mitochondrial quality control and preserving MAM homeostasis through regulation of GRP75 acetylation. These findings identify the SIRT1–GRP75/MAM axis as a potential therapeutic target for preventing oxidative stress-induced pelvic floor tissue dysfunction.

## 1 Introduction

Stress urinary incontinence (SUI) represents the most prevalent form of urinary incontinence among women, characterized by the involuntary leakage of urine prompted by increased intra-abdominal pressure during activities such as coughing, sneezing, laughing, or physical exertion. This condition can substantially diminish quality of life, particularly in postpartum and elderly women, with reported prevalence rates of 18% and 34%, respectively (de Macedo Dantas et al., 2019; Jurášková et al., 2020). The normal functionality of female pelvic organs is contingent upon an intact anatomical structure, encompassing pelvic muscles, nerves, and connective tissue. The anterior vaginal wall plays a pivotal role in sustaining the normal positioning and function of the bladder neck and urethra, to which it is attached (Ashton-Miller and DeLancey, 2007; DeLancey, 1994). A notable pathological alteration in the vaginal wall following the onset of SUI is the disruption of collagen metabolism and apoptosis within the anterior vaginal wall, attributed to oxidative stress. Research has demonstrated that the administration of antioxidants and the overexpression of Nrf2 can effectively ameliorate SUI in a murine model (Habes et al., 2022; Hong et al., 2015; Tang et al., 2017). Consequently, mitigating oxidative stress has emerged as a critical objective in the amelioration of SUI symptoms.

Mitochondrial metabolism is responsible for the production of reactive oxygen species (ROS), which, under physiological conditions, serve as intracellular signaling molecules that regulate various cellular processes. Nonetheless, tissue damage can result in the accumulation of ROS, thereby inducing oxidative stress (Kattoor et al., 2017). An excessive buildup of ROS leads to both functional impairments in the electron transport chain and structural anomalies in mitochondria, ultimately activating the mitochondria-mediated intrinsic apoptosis pathway and culminating in cell death (Sinha et al., 2013; Zhang et al., 2022). The regulation of mitophagy and mitochondrial biogenesis is essential for the preservation of mitochondrial function (Li et al., 2021a; Su et al., 2023). Damaged and dysfunctional mitochondria are selectively degraded and recycled through the coordinated action of key proteins, including PINK1, parkin, p62, and LC3B(Lu et al., 2023). In contrast, mitochondrial biogenesis encompasses the generation of new, functional mitochondria to satisfy cellular energy requirements and sustain normal cellular function (Liu et al., 2023; Zhao et al., 2023). Oxidative stress is frequently associated with an influx of calcium into mitochondria, which leads to the production of additional reactive oxygen species (ROS), thereby intensifying oxidative stress (Gou et al., 2024). Mitochondria and the endoplasmic reticulum are interlinked, with their contact sites forming mitochondria-associated membranes (MAMs) that facilitate the transfer of calcium from the endoplasmic reticulum to the mitochondria (Ni and Yuan, 2021).

SIRT1, a nicotinamide adenine dinucleotide (NAD)-dependent histone deacetylase, is essential for cellular mitochondrial function and network remodeling, playing a crucial role in the regulation of oxidative stress and cellular senescence (Oanh et al., 2017; Sun et al., 2022; Yan et al., 2021). It exerts its influence on mitochondrial homeostasis through its deacetylase activity (Chandrasekaran et al., 2019; Oanh et al., 2017; Wang et al., 2023). In individuals with pelvic organ prolapse (POP), SIRT1 activity is inhibited in the fibroblasts of the uterosacral ligament, and enhancing SIRT1 activity has been demonstrated to mitigate oxidative stress (Wang et al., 2024). Nevertheless, the specific mechanisms through which SIRT1 maintains mitochondrial homeostasis to counteract oxidative stress remain inadequately understood.

Ginsenosides, the principal bioactive constituents of ginseng, encompass ginsenoside Rb1, which is the most abundant. Ginsenoside Rb1 has exhibited antioxidant properties and advantageous effects on a range of pathological conditions (Jiang et al., 2021). It modulates mitochondrial function via pathways related to mitochondrial biogenesis, energy metabolism, oxidative stress response, and mitophagy (Cui et al., 2017; Sun et al., 2013; Zheng et al., 2017; Zhou et al., 2022). Despite the growing elucidation of Rb1’s mechanisms in the management of certain diseases, its potential benefits and underlying mechanisms in the treatment of stress urinary incontinence (SUI) remain inadequately understood.

In this study, we examined the protective effects and molecular mechanisms of Rb1 in a mouse model of SUI induced by vaginal distension (VD) and a reductionist H_2_O_2_-induced oxidative stress model in L929 cells. Our findings revealed that Rb1 enhanced urodynamic function, maintained collagen organization, and mitigated oxidative damage in the anterior vaginal wall tissues. Mechanistically, Rb1 upregulated SIRT1 and enhanced mitochondrial biogenesis-associated signaling through the PGC-1α/NRF1/TFAM pathway, while promoting PINK1/Parkin-associated mitophagic signaling. Additionally, Rb1 preserved mitochondria-associated membrane (MAM) integrity by regulating SIRT1-associated GRP75 deacetylation, thereby reducing excessive endoplasmic reticulum–mitochondrial calcium transfer, mitochondrial calcium overload, and mtROS accumulation. These findings highlight the SIRT1–GRP75/MAM axis as a potential mechanism underlying Rb1-mediated protection against SUI and provide new insights into mitochondrial homeostasis-based therapeutic strategies for pelvic floor disorders (Figure 1).

**FIGURE 1:**
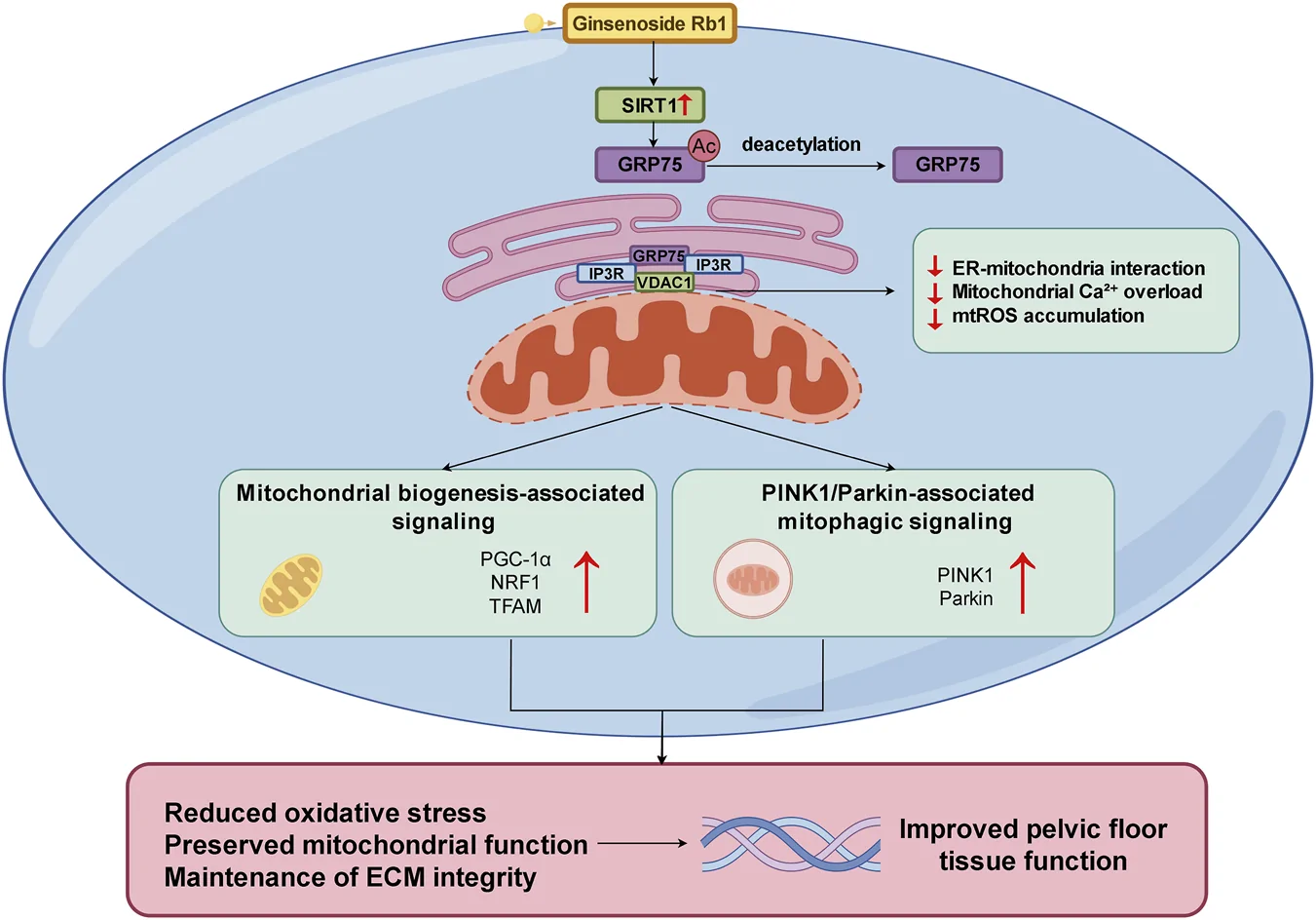
Schematic illustration of the protective mechanism of ginsenoside Rb1 against stress urinary incontinence through SIRT1‐mediated mitochondrial homeostasis and MAM integrity restoration. By Figdraw.

## 2 Materials and methods

### 2.1 Animal experimental design

Female C57BL/6 mice aged 10 weeks were used in this study. All animal procedures were reviewed and approved by the Institutional Animal Care and Ethics Committee of the People’s Hospital of Wuhan University and performed in accordance with institutional guidelines (WDRM20200805). Animals were randomly allocated to five experimental groups: control (CON), vaginal distension (VD), VD with low-dose Rb1 treatment (VD + Rb1-L, 10 mg/kg/day), VD with high-dose Rb1 treatment (VD + Rb1-H, 20 mg/kg/day), and VD with high-dose Rb1 combined with the SIRT1 inhibitor EX527 (VD + Rb1+EX527, 20 mg/kg/day Rb1 and 5 mg/kg/day EX527). Ginsenoside Rb1 (Shanghai Yanyi Bio-Technology Co., Ltd., Shanghai, China) was prepared in DMSO and administered intraperitoneally once daily for seven consecutive days beginning on the first day after VD. EX527 was administered intraperitoneally at 5 mg/kg/day concurrently with Rb1 in the combination group. Equivalent amounts of DMSO were given to the CON and VD groups. The SUI model was generated using a previously established simulated vaginal delivery procedure (Yang et al., 2023). On day 7 after VD, bladder leak point pressure (BLPP) was determined to confirm successful model establishment and assess the therapeutic effects of Rb1. Following urodynamic evaluation, the mice were euthanized and the anterior vaginal wall tissues were harvested for subsequent analyses.

### 2.2 Cell culture and treatments

The murine fibroblast cell line L929 was provided by the China Center for Type Culture Collection (Wuhan, China). Cells were grown in MEM (Wuhan Pricella Biotechnology Co., Ltd.) containing 10% FBS (Gemini Bio-Products, CA, United States) and 1% double antibiotics (100 KU/ml penicillin G and 100 mg/mL streptomycin; Genom Biotech Ltd., Hangzhou, China). Cell cultures were maintained at 37 °C under a humidified atmosphere containing 5% CO_2_, with the culture medium replaced every 2 days. Cells were subcultured or cryopreserved when approximately 70% confluence was reached. For oxidative stress induction, exponentially growing L929 cells were exposed to H_2_O_2_ (Sigma-Aldrich, St. Louis, MO, United States) for 4 h. This H_2_O_2_ exposure paradigm was used as a reductionist model of oxidative stress to investigate redox-dependent cellular mechanisms. Where indicated, cells subsequently received Rb1 treatment at 20 or 40 μM for 24 h. For pharmacological inhibition of SIRT1, cells were treated with Rb1 together with EX527 (10 μM) for 24 h following H_2_O_2_ exposure.

### 2.3 siRNA transfection

For SIRT1 silencing, L929 cells were plated in 6-well culture plates and transfected with SIRT1-targeting siRNAs using InvitroRNA™ according to the manufacturer’s instructions. The siRNA oligonucleotides were synthesized by Genepharma Co., Ltd. (Suzhou, China). Three independent siRNAs targeting SIRT1 were initially evaluated for their knockdown efficiency by Western blotting, using a non-targeting siRNA (si-NC) as the negative control (Supplementary Figure S1A). The si-NC sequence was as follows: forward, UUC​UCC​GAA​CGU​GUC​ACG​UTT; reverse, ACG​UGA​CAC​GUU​CGG​AGA​ATT. All three SIRT1-targeting siRNAs effectively reduced SIRT1 expression, and the siRNA showing the most efficient knockdown was selected for subsequent experiments. The sequence used for SIRT1 knockdown in the subsequent experiments was as follows: forward, GCA​CCG​AUC​CUC​GAA​CAA​UTT; reverse, AUU​GUU​CGA​GGA​UCG​GUG​CTT. The si-NC was used only for the initial validation of SIRT1 knockdown efficiency and was not included in the subsequent mechanistic experiments. Unless otherwise indicated, cells were subjected to the subsequent experimental treatments after siRNA transfection.

### 2.4 Western blot analysis

Protein lysates were prepared from cultured L929 cells and anterior vaginal wall specimens using RIPA buffer supplemented with PMSF (Servicebio, Wuhan, China). Protein concentrations were quantified with a BCA protein assay kit (Beyotime Institute of Biotechnology, Haimen, China). Equivalent quantities of protein were resolved by 10% SDS-PAGE and electrotransferred onto 0.45-μm PVDF membranes (Pall, Port Washington, NY, United States). Membranes were blocked with 5% skim milk and subsequently incubated at 4 °C overnight with primary antibodies against SIRT1 (1:1,000), PGC-1α (1:1,000), NRF1 (1:5,000), TFAM (1:5,000), COXIV (1:1,000), PINK1 (1:2000), Parkin (1:2000), collagen I (1:1,000), collagen III (1:1,000), cytochrome c (1:1,000), Bcl2 (1:1,000), BAX (1:1,000), Nrf2 (1:1,000), GRP75 (1:5,000), FUNDC1 (1:1,000, Cell Signaling Technology, Danvers, MA, United States), and β-actin (1:20,000). All antibodies were obtained from Proteintech Group, Inc., Rosemont, IL, United States unless otherwise specified. After incubation with the primary antibodies, membranes were washed and exposed to HRP-conjugated goat anti-mouse or goat anti-rabbit IgG for 1 h at room temperature. Protein signals were developed with West Pico PLUS enhanced chemiluminescent substrate (Bioleki Biotechnology Co., Ltd., Hubei, China) and recorded using an imaging system (Servicebio, Wuhan, China).

### 2.5 Measurement of mitochondrial reactive oxygen species

Mitochondrial ROS production was assessed using MitoSOX™ Red (Invitrogen, Carlsbad, CA, United States). Following the indicated treatments, L929 cells were collected by trypsinization and incubated with 5 μM MitoSOX™ Red for 30 min at 37 °C. The stained cells were subsequently washed twice with PBS and resuspended in PBS before flow cytometric examination. Fluorescence was acquired on a CytoFLEX flow cytometer (Beckman Coulter, Brea, CA, United States) using excitation and emission wavelengths of 510 and 580 nm, respectively. The mean fluorescence intensity (MFI) was quantified with FlowJo software version 10 (TreeStar Inc., Ashland, OR, United States).

### 2.6 Assessment of mitochondrial membrane potential

Changes in mitochondrial membrane potential (MMP) were evaluated using a JC-1 assay kit (Beyotime Biotechnology, Shanghai, China). After completion of the indicated treatments, L929 cells were harvested and incubated with JC-1 for 30 min at 37 °C. The fluorescence signals corresponding to JC-1 monomers and aggregates were subsequently measured by flow cytometry using a CytoFLEX system (Beckman Coulter, Brea, CA, United States). The red and green fluorescence signals were detected at Ex/Em = 485/590 nm. MMP was represented by the ratio of red to green fluorescence and expressed relative to the untreated control group.

### 2.7 Determination of intracellular ROS

Intracellular ROS generation was determined using a Reactive Oxygen Species Assay Kit (Beyotime Biotechnology, Shanghai, China). Briefly, treated L929 cells were collected and incubated with DCFH-DA for 20 min at 37 °C. Cells were then washed three times with PBS to remove excess fluorescent probe and resuspended in flow buffer. Fluorescence was recorded using a CytoFLEX flow cytometer (Beckman Coulter, Brea, CA, United States) at an excitation wavelength of 488 nm and an emission wavelength of 525 nm. The resulting MFI values were obtained using FlowJo software version 10 (TreeStar Inc., Ashland, OR, United States).

### 2.8 Quantification of mitochondrial DNA

Total DNA was isolated from L929 cells and anterior vaginal wall tissues with the MolPure Cell/Tissue DNA Kit (Yeasen Biotechnology (Shanghai) Co., Ltd., Shanghai, China). The relative mitochondrial DNA content was evaluated by quantifying the mitochondrial gene mtAtp6 and the nuclear reference gene Tert using RT-qPCR. Primer sequences are provided in Supplementary Table S1 and were synthesized by Sangon Biotech (Shanghai, China). The relative abundance of mtDNA was calculated from the expression ratio of mtAtp6 to Tert.

### 2.9 Cell viability analysis

The effects of Rb1 and H_2_O_2_ treatment on L929 cells viability were examined using the SuperKine™ Maximum Sensitivity Cell Counting Kit-8 (CCK-8; Abbkine Scientific Co., Ltd., Wuhan, China). Cells were plated in 96-well plates 1 day before treatment. After exposure to the indicated concentrations of Rb1 and H_2_O_2_, cells were washed three times with PBS. Subsequently, 10 μL of culture medium and 10 μL of CCK-8 reagent were added to each well, followed by incubation at 37 °C for 1–2 h. Absorbance was recorded at 450 nm using a microplate reader. Cell viability was expressed as a percentage of the untreated control.

### 2.10 Masson’s trichrome staining

Paraffin-embedded anterior vaginal wall tissues were sectioned at a thickness of 4 μm and processed using a Masson Stain Kit (HT15, Sigma, United States) following the manufacturer’s recommended procedure. Collagen fibers were visualized as blue-stained structures. The collagen-positive area was quantified by calculating the mean optical density (MOD) using ImageJ software (NIH, Bethesda, MD, United States).

### 2.11 Transmission electron microscopy

For ultrastructural analysis, anterior vaginal wall specimens were trimmed into approximately 0.8–1.0 mm^3^ pieces and immediately immersed in 2.5% pre-cooled glutaraldehyde at 4 °C for 6 h. The specimens were subsequently dehydrated through a graded ethanol series and acetone, followed by infiltration with a 1:1 mixture containing propylene oxide and resin and embedding in resin. Ultrathin sections approximately 50 nm thick were prepared, mounted on grids, and sequentially stained with 4% uranyl acetate for 15–30 min and 0.5% lead citrate for 3–15 min. Ultrastructural features were examined using a transmission electron microscope (Hitachi Ltd., Tokyo, Japan). MAM coverage and the distance between mitochondria and the endoplasmic reticulum were quantified on TEM images using ImageJ, as previously described (Lam et al., 2021).

For quantitative TEM analysis, three randomly selected TEM fields were examined for each biological replicate in cell culture experiments and for each mouse in animal experiments. All clearly identifiable mitochondria within the selected fields were included for quantitative analysis. MAM parameters, including MAM distance and contact length, were measured using ImageJ. Measurements obtained from multiple mitochondria within the same biological replicate or mouse were averaged to generate a single value for each biological replicate or mouse, respectively, which was considered the experimental unit for statistical analysis.

### 2.12 Immunohistochemical staining

Paraffin sections of anterior vaginal wall tissue were deparaffinized and rehydrated before immunohistochemical analysis. Sections were treated with 10% goat serum to reduce nonspecific antibody binding and then incubated overnight at 4 °C with primary antibodies recognizing PGC-1α (1:150), NRF1 (1:50), TFAM (1:200), 4-HNE (1:200), and 8-OHdG (1:200). Following removal of unbound primary antibodies by washing, sections were incubated with biotin-conjugated goat anti-rabbit/mouse IgG (1:500) for 2 h at room temperature. Streptavidin-biotin HRP complexes were subsequently applied, and immunoreactivity was visualized using a pre-concentrated DAB chromogenic substrate. Images were acquired using an upright microscope (BX53, Olympus, Tokyo, Japan), and staining intensity quantified with ImageJ.

For quantitative immunohistochemical analysis, three randomly selected fields were examined for each mouse. Quantitative analysis of staining intensity was performed using ImageJ. Measurements obtained from multiple fields within the same mouse were averaged to generate a single value for each mouse, which was considered the experimental unit for statistical analysis.

### 2.13 TUNEL staining

Cell apoptosis in the anterior vaginal wall was examined using a TUNEL assay with an *In Situ* Cell Death Detection Kit (Roche, Mannheim, Germany). Tissue sections were processed in accordance with the manufacturer’s instructions. TUNEL-positive cells were identified by brown granular signals predominantly localized within the nuclei. The proportion of apoptotic cells was determined by dividing the number of TUNEL-positive cells by the total number of cells, with quantitative analysis performed using ImageJ software.

### 2.14 Immunofluorescence staining

For cellular immunofluorescence analysis, L929 cells were cultured in 6-well plates and subjected to the indicated H_2_O_2_, Rb1, and SIRT1 knockdown treatments. Cells were fixed with 4% formaldehyde for 10 min, permeabilized with 0.3% Triton X-100 for 10 min, and blocked with 5% BSA for 30 min at room temperature. For tissue immunofluorescence, anterior vaginal wall specimens were harvested 1 day after completion of the 7-day VD procedure following perfusion with ice-cold PBS. The tissues were fixed in 4% paraformaldehyde for 48 h and subsequently sectioned. Tissue sections were permeabilized with 0.3% Triton X-100 for 10 min and blocked with 5% FBS for 60 min at room temperature. Both cell and tissue samples were incubated overnight at 4 °C with primary antibodies against PGC-1α, NRF1, TFAM, IP3R, PINK1, and Parkin (1:100). After washing, appropriate Cy3- or FITC-conjugated goat anti-rabbit or anti-mouse IgG secondary antibodies (1:200; Servicebio, Wuhan, China) were applied for 1 h in the dark at room temperature. Mitochondria were labeled using Mito-Tracker, whereas nuclei were counterstained with DAPI. Fluorescence images were obtained using an upright microscope (BX53, Olympus, Tokyo, Japan) and a confocal laser scanning microscope (FV1200, Olympus, Tokyo, Japan). ImageJ was used for subsequent fluorescence quantification and image analysis.

For quantitative immunofluorescence analysis, three randomly selected fields were examined for each biological replicate in cell culture experiments and for each mouse in animal experiments. Quantitative analysis of fluorescence intensity and colocalization was performed using ImageJ, as appropriate. Measurements obtained from multiple fields within the same biological replicate or mouse were averaged to generate a single value for each biological replicate or mouse, respectively, which was considered the experimental unit for statistical analysis.

### 2.15 Mitochondrial Ca^2+^ measurement

Mitochondrial Ca^2+^ levels in L929 cells were determined using the cell-permeable fluorescent probe Rhod-2/AM (Yeasen Biotechnology (Shanghai) Co., Ltd., Shanghai, China). Briefly, cells cultured in 6-well plates were washed three times with an appropriate buffer after removal of the culture medium. Rhod-2/AM was prepared at a working concentration of 2 μM and added to the cells, followed by incubation at 37 °C for 30 min. After incubation, the staining solution was removed and the cells were washed three times with buffer to eliminate excess probe. Fresh buffer was subsequently added to cover the cells, followed by an additional incubation at 37 °C for 20–30 min to allow intracellular de-esterification of the probe. Fluorescence was then visualized using an inverted microscope (X71, Olympus, Tokyo, Japan), and the fluorescence intensity was quantified using ImageJ software.

### 2.16 Molecular docking analysis

The potential interaction between ginsenoside Rb1 and SIRT1 was investigated using molecular docking analysis. The SDF format structure of ginsenoside Rb1 was retrieved from the PubChem database, while the three-dimensional structure of SIRT1 was obtained from the Protein Data Bank (PDB). Before docking, the SIRT1 structure was prepared using PyMOL 2.6.0 by removing solvent molecules. Further structural preparation, including hydrogen atom addition and charge assignment, was performed with AutoDock Tools. Both the ligand and receptor were subsequently converted to PDBQT format. An appropriate docking grid was defined according to the predicted binding region, and molecular docking was performed using AutoDock Vina 1.2.3. The calculated binding energy was used to evaluate the predicted interaction between Rb1 and SIRT1, with more negative values indicating energetically more favorable binding. Binding energies of ≤−5.0 kcal/mol and ≤−7.5 kcal/mol were considered indicative of good and very strong binding interactions, respectively. The predicted binding mode and ligand–protein interactions were visualized using PyMOL 2.6.0 and Discovery Studio, including two-dimensional interaction maps.

### 2.17 Co-immunoprecipitation and acetylation analysis

To evaluate the acetyl-lysine-associated signals of MAM-associated proteins, L929 cells were subjected to the indicated treatments and lysed using an appropriate ice-cold lysis buffer containing protease and deacetylase inhibitors. Equal amounts of total protein were incubated with an anti-acetylated lysine Ac-K antibody overnight at 4 °C with gentle rotation. Protein A/G agarose beads were subsequently added and incubated for an additional 2–4 h at 4 °C. The beads were washed several times with cold lysis buffer, and the immunoprecipitated proteins were eluted by boiling in SDS sample buffer. The immunoprecipitates were subjected to Western blotting and analyzed using antibodies against GRP75, MFN2, or FUNDC1 to assess their acetyl-lysine-associated signals. Corresponding input samples were analyzed by Western blotting to determine the total protein abundance of each target protein. The relative acetyl-lysine-associated signal was calculated by normalizing the immunoprecipitated target protein signal to its corresponding input protein level, followed by normalization to the control group.

To investigate the interaction between SIRT1 and GRP75, reciprocal co-immunoprecipitation assays were performed. Briefly, equal amounts of cellular lysates were incubated overnight at 4 °C with an anti-SIRT1 or anti-GRP75 antibody, followed by incubation with protein A/G agarose beads. After extensive washing, the immunoprecipitated proteins were eluted and subjected to Western blotting with antibodies against GRP75 or SIRT1, respectively. Normal IgG was used as a negative immunoprecipitation control, and input lysates were analyzed in parallel to verify the abundance of the target proteins.

### 2.18 Construction of GRP75 acetylation mutants

To investigate the functional role of GRP75 acetylation, GRP75 acetylation mutants were generated based on previously reported acetylation sites at lysine residues K567 and K612. These sites were selected according to previous studies demonstrating that GRP75-K567/612 acetylation regulates the interaction between GRP75 and IP3R and modulates ER–mitochondrial calcium communication (Wang et al., 2025). The wild-type GRP75 construct GRP75-WT and two acetylation-state mimetic mutants, GRP75-2KR and GRP75-2KQ, were generated by site-directed mutagenesis. In the GRP75-2KR construct, K567 and K612 were substituted with arginine K567R/K612R to mimic the deacetylated state, whereas the same lysine residues were substituted with glutamine K567Q/K612Q in the GRP75-2KQ construct to mimic the acetylated state. The resulting constructs were verified by sequencing and subsequently introduced into L929 cells using an appropriate transfection method. Cells expressing GRP75-WT, GRP75-2KR, or GRP75-2KQ were used for subsequent acetylation, MAM morphology, protein colocalization, and mitochondrial calcium analyses.

### 2.19 Assessment of GRP75 acetyl-lysine-associated signal

To assess the acetyl-lysine-associated signal of GRP75-WT, GRP75-2KR, and GRP75-2KQ, GRP75-expressing cells were lysed, and GRP75-associated acetyl-lysine signal was assessed by immunoprecipitation using an anti-Ac-K antibody, followed by Western blotting with an anti-GRP75 antibody. The acetyl-lysine-associated signal of GRP75 was quantified after normalization to the corresponding input GRP75 protein level.

### 2.20 Statistical analysis

All statistical analyses were performed using GraphPad Prism 9.0 (GraphPad Software Inc., San Diego, CA, United States). Quantitative data are presented as mean ± standard deviation (SD). For cell culture experiments, n represents the number of independent biological experiments, whereas for animal experiments, n represents the number of animals per group. The exact sample size for each experiment is indicated in the corresponding figure legends.

Multiple microscopic fields, cells, or mitochondria obtained from the same biological replicate were considered technical observations rather than independent biological replicates. Multiple measurements within each biological replicate were averaged to generate a single value for that biological replicate, which was used as the statistical unit for subsequent analyses to avoid pseudoreplication.

Animals were randomly allocated to the experimental groups after acclimatization. Investigators performing histological, immunohistochemical, fluorescence, and TEM image quantification were blinded to group allocation, where applicable. Before statistical analysis, data were assessed for approximate normality and homogeneity of variance to verify the assumptions of parametric tests.

Comparisons between two groups were performed using an unpaired two-tailed Student’s t-test when appropriate. Comparisons among multiple groups were performed using one-way analysis of variance (ANOVA) followed by Dunnett’s multiple-comparison test, with the reference group specified in the corresponding figure legend.

For figures containing both animal and cell experiments, statistical analyses were performed separately within each experimental system using the corresponding biological replicate as the statistical unit and the reference group indicated in the figure legend. The meanings of the statistical symbols (*, #, and &) and their corresponding reference groups are specified in each figure legend. Statistical significance was defined as P < 0.05.

## 3 Results

### 3.1 Ginsenoside Rb1 attenuates collagen loss and urodynamic disturbances after VD in mice

In this study, we investigated the effects of ginsenoside Rb1 (Figure 2A) on collagen loss and urodynamic disturbances in a SUI mouse model induced by VD. Following VD, ginsenoside Rb1 was administered intraperitoneally at low (10 mg/kg) and high (20 mg/kg) concentrations for seven consecutive days. On the seventh day, we measured BLPP, a critical urodynamic parameter, and assessed histopathological changes in the anterior vaginal wall of the treated mice (Figure 2B). Our analyses revealed a statistically significant reduction in BLPP levels in the VD group compared to the control group. However, administration of ginsenoside Rb1 effectively prevented the VD-induced decline in BLPP, with higher concentrations of the compound yielding more pronounced improvements in urodynamic parameters (Figure 2C). Further examination of the anterior vaginal wall showed that Rb1 significantly alleviated the VD-induced disruption of collagen fiber arrangement and reduced collagen expression (Figures 2D,E). Additionally, Rb1 was effective in reducing apoptosis in the anterior vaginal wall cells of VD mice (Figures 2F,G). These findings suggest that ginsenoside Rb1 exerts a protective effect against VD-induced collagen metabolism disorders and urodynamic dysregulation in the anterior vaginal wall of mice.

**FIGURE 2:**
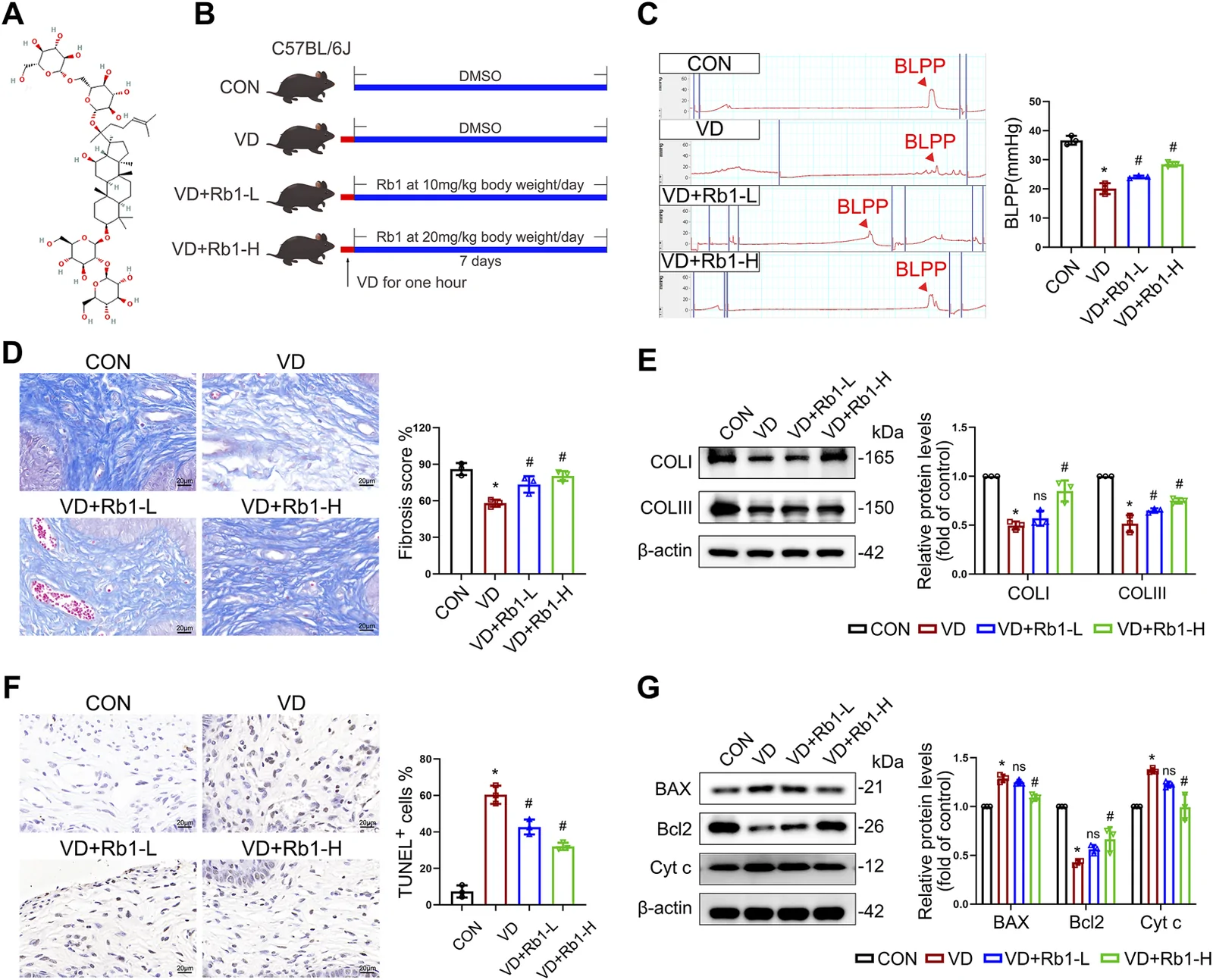
Ginsenoside Rb1 improves urodynamics and collagen metabolism disorders in the SUI mice anterior vaginal wall. **(A)** Chemical structure of ginsenoside Rb1. **(B)** Experimental design of VD mice treated with different concentrations of ginsenoside Rb1. **(C)** The represent-curve of urinary dynamics of different groups, and statistical analysis of BLPP for different groups on 7 days. **(D)** Masson staining for collagen expression in the tissue of the anterior vaginal wall in mice of four groups. **(E)** Immunoblot analysis of collagen I (COL I) and collagen III (COL III) in anterior vaginal wall in mice. Quantification represents the level of the indicated protein normalized to β-actin. **(F)** The detection of apoptosis in the anterior vaginal wall tissue of four groups was conducted using a TUNEL kit. **(G)** Immunoblot analysis of BAX, Bcl2 and Cyt c in anterior vaginal wall in mice. Quantification represents the level of the indicated protein normalized to β-actin. Data are expressed as mean ± SD. For all panels, n = 3 mice per group. *P < 0.05 compared with the control group, ^#^ *P* < 0.05 compared with the VD group, and ns indicates no significant difference.

### 3.2 Ginsenoside Rb1 alleviates oxidative stress *in vitro* and *in vivo*

Recent research underscores the significant role of oxidative stress in the etiology of pelvic floor dysfunction disorders. In our study, we investigated oxidative stress markers in the anterior vaginal wall of mice. We found that the lipid oxidative damage marker 4-HNE and the DNA oxidative damage marker 8-OHdG, which showed increased expression following VD, exhibited decreased expression after treatment with ginsenoside Rb1 (Figures 3A,B). To investigate redox-dependent cellular mechanisms associated with the protective effects of Rb1, we further used H_2_O_2_-treated L929 fibroblasts as a reductionist oxidative stress model. The results showed that Rb1 enhanced cell viability under oxidative stress within the concentration range of 20–80 μM (Figure 3C). Therefore, low concentration (20 μM) and high concentration (40 μM) of Rb1 were selected for further validation. Further analysis revealed a significant reduction in intracellular ROS levels following Rb1 treatment (Figure 3D). Moreover, the antioxidant protein Nrf2 was significantly elevated after Rb1 treatment (Figure 3E). In conclusion, ginsenoside Rb1 effectively mitigates oxidative stress both *in vivo* and *in vitro*.

**FIGURE 3:**
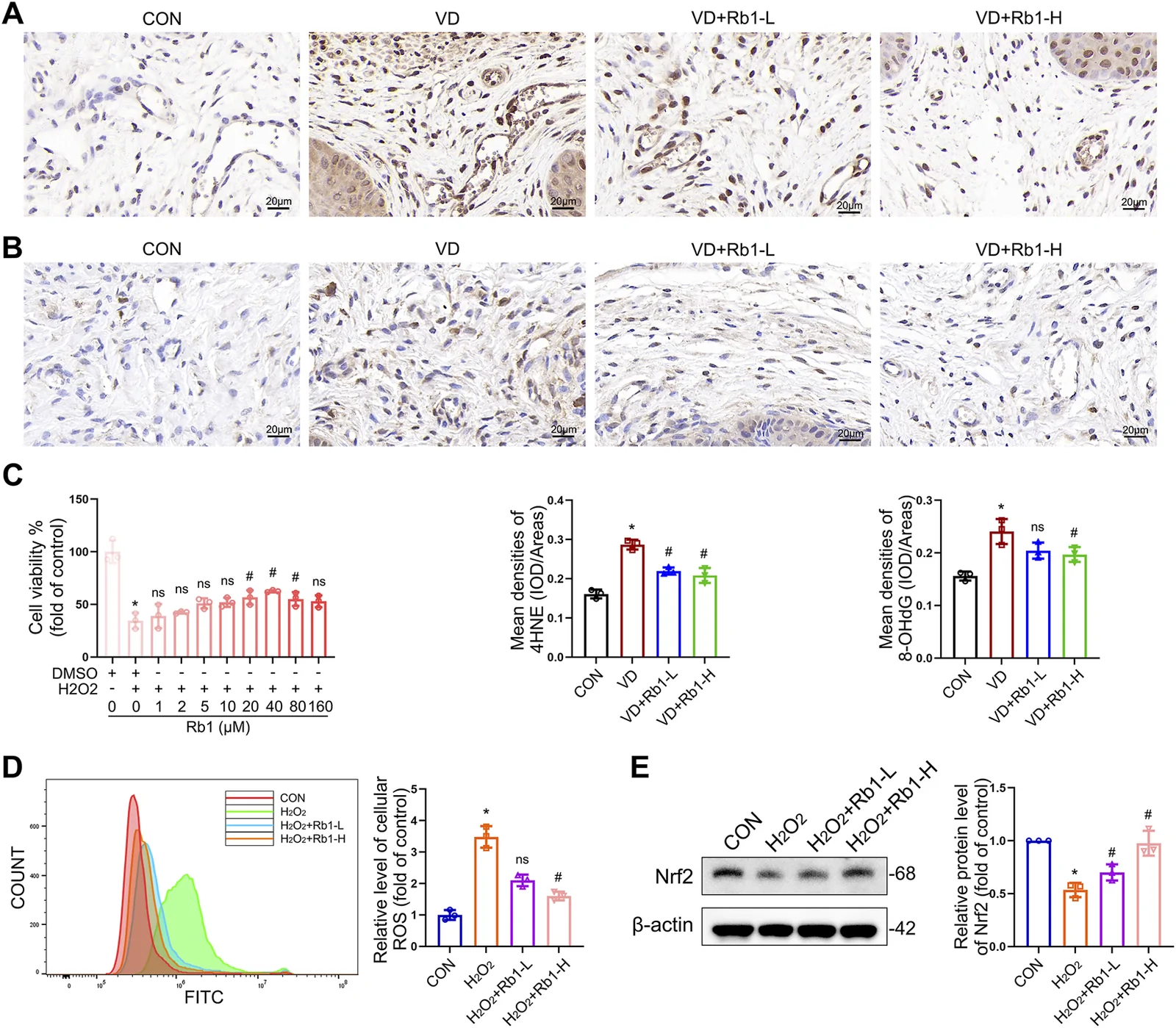
Ginsenoside Rb1 ameliorates oxidative damage in the anterior vaginal wall of SUI mice. **(A,B)** Representative images of the IHC staining for 4-HNE **(A)** and 8-OHdG **(B)** in anterior vaginal wall were obtained and quantified. **(C)** The viability level of L929 cells was determined by CCK-8 assay. L929 cells were treated with 1, 2, 5, 10, 20, 40, 80, 160 µM Rb1 or DMSO for 24 h after H_2_O_2_ treatment. **(D)** Representative flow plots of ROS production in L929 cells measured by flow cytometry. **(E)** Immunoblot analysis of Nrf2 in L929 cells. Quantification represents the levels of the indicated protein normalized to β-actin. Data are expressed as mean ± SD. For animal experiments **(A,B)**, n = 3 mice per group. For cell experiments **(C–E)**, n = 3 independent experiments. For **(A,B)**, *P < 0.05 compared with the control group and ^#^ *P* < 0.05 compared with the VD group. For **(C–E)**, *P < 0.05 compared with the control group and ^#^ *P* < 0.05 compared with the H_2_O_2_ group. ns indicates no significant difference.

### 3.3 Ginsenoside Rb1 enhances mitochondrial-biogenesis-associated signaling

Oxidative stress is strongly associated with mitochondrial damage, and mitochondrial biogenesis plays a crucial role in maintaining mitochondrial populations by replacing damaged mitochondria (Ho and Shirakawa, 2022). This biogenesis is regulated by both mitochondrial and nuclear genes and is controlled by multiple transcription factors (Jiang et al., 2018; Qian et al., 2019). In our study, we investigated the expression of PGC-1α, NRF1, and TFAM and found that Rb1 enhanced PGC-1α/NRF1/TFAM-associated signaling, which was suppressed by H_2_O_2_ (Figure 4A). Immunofluorescence analysis further indicated that Rb1 enhanced PGC-1α/NRF1/TFAM-associated signaling (Supplementary Figures S1B–D). Microscopic observation showed that H_2_O_2_ treatment disrupted mitochondrial structure, while Rb1 helped maintain normal mitochondrial morphology (Figure 4B). Furthermore, our study found that Rb1 effectively increased the levels of the mitochondrial protein COXIV and the content of mtDNA (Figures 4C,D). To further substantiate the beneficial impact of Rb1 on mitochondrial biogenesis-associated signaling, we examined the anterior vaginal wall of VD mice following ginsenoside Rb1 treatment. The results demonstrated that the PGC-1α/NRF1/TFAM axis, which was inhibited in the anterior vaginal wall of VD mice, was activated following Rb1 treatment (Figure 4E). Moreover, the mtDNA content in the anterior vaginal wall was augmented in mice treated with Rb1 (Figure 4F). These findings suggest that ginsenoside Rb1 enhances a mitochondrial biogenesis-associated program through the PGC-1α/NRF1/TFAM pathway.

**FIGURE 4:**
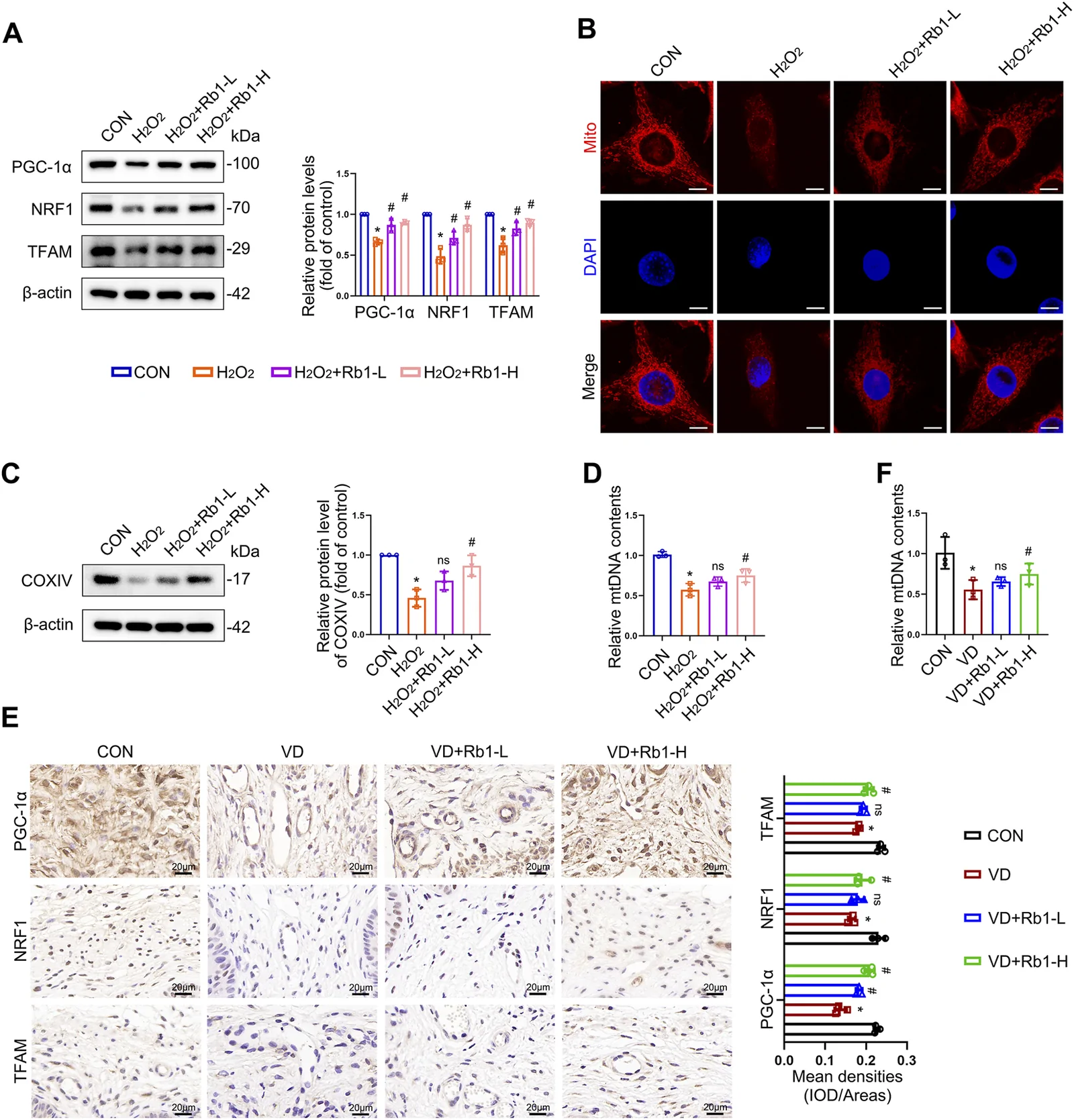
Ginsenoside Rb1 promotes mitochondrial biogenesis-associated signaling. **(A)** Immunoblot analyses of PGC-1α, NRF1 and TFAM in L929 cells. Quantification represents the levels of the indicated protein normalized to β-actin. **(B)** Representative images from Mito-tracker-stained control and H_2_O_2_-treated L929 cells with or without Rb1 treatment. Scale bars = 10 μm. **(C)** Immunoblot analysis of COXIV in L929 cells. Quantification represents the level of the indicated protein normalized to β-actin. **(D)** RT-qPCR analyses of relative mtDNA contents in L929 cells. **(E)** Representative images of the IHC staining for PGC-1α, NRF1 and TFAM in anterior vaginal wall were obtained and quantified. **(F)** RT-qPCR analyses of relative mtDNA contents in the anterior vaginal wall of mice. Data are expressed as mean ± SD. For cell culture experiments **(A–D)**, n = 3 independent experiments. For animal experiments **(E,F)**, n = 3 mice per group. For **(A–D)**, *P < 0.05 compared with the control group and ^#^ *P* < 0.05 compared with the H_2_O_2_ group. For **(E,F)**, *P < 0.05 compared with the control group and ^#^ *P* < 0.05 compared with the VD group. ns indicates no significant difference.

### 3.4 Ginsenoside Rb1 promotes PINK1/parkin-associated mitophagic signaling

PINK1/Parkin-associated mitophagic signaling plays an important role in mitochondrial quality control and has been implicated in the removal of damaged mitochondria. Our study demonstrated that exposure to H_2_O_2_ reduced the expression of PINK1 and Parkin, whereas ginsenoside Rb1 enhanced PINK1/Parkin-associated signaling (Figure 5A). Further results showed that H_2_O_2_ treatment reduced the recruitment of PINK1 to mitochondria, whereas Rb1 restored PINK1 mitochondrial recruitment (Figure 5B). Moreover, Rb1 promoted the translocation of Parkin to mitochondria (Figure 5C). This hypothesis was further validated in the anterior vaginal wall of mice, where the expression of PINK1 and parkin was reduced following VD, but this reduction was counteracted by Rb1 (Figures 5D,E). Collectively, these findings suggest that ginsenoside Rb1 enhances PINK1/Parkin-associated mitophagic signaling and mitochondrial quality-control responses, which may contribute to the maintenance of mitochondrial homeostasis.

**FIGURE 5:**
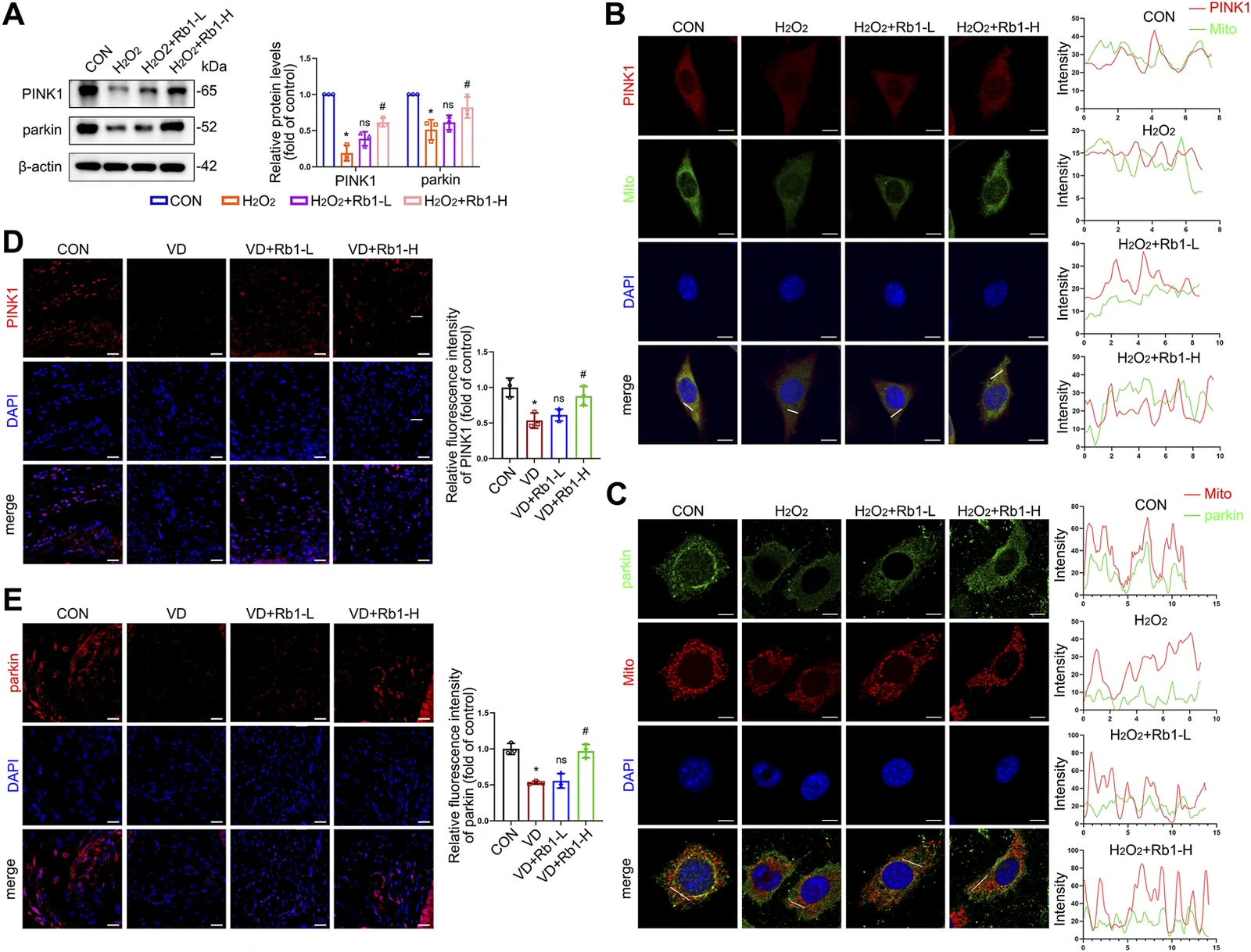
Ginsenoside Rb1 promotes PINK1/Parkin-associated mitophagic signaling. **(A)** Immunoblot analyses of PINK1 and parkin in L929 cells. Quantification represents the levels of the indicated protein normalized to β-actin. **(B,C)** Colocalization analysis of immunofluorescence images of PINK1 (red) and Mito-tracker (green) **(B)**, parkin (green) and Mito-tracker (red) **(C)** in different groups of L929 cells after treated with H_2_O_2_ with or without different concentrations of Rb1. Scale bar = 10 μm. **(D,E)** Immunofluorescence staining of PINK1 **(D)** and parkin **(E)** in the anterior vaginal wall of mice. Scale bar = 20 μm. Data are expressed as mean ± SD. For cell culture experiments **(A–C)**, n = 3 independent experiments. For animal experiments **(D,E)**, n = 3 mice per group. For **(A–C)**, *P < 0.05 compared with the control group and ^#^ *P* < 0.05 compared with the H_2_O_2_ group. For **(D,E)**, *P < 0.05 compared with the control group and ^#^ *P* < 0.05 compared with the VD group. ns indicates no significant difference.

### 3.5 Ginsenoside Rb1 alleviates oxidative stress by maintaining MAMs homeostasis, alleviating mitochondrial calcium overload and reducing mitochondrial ROS accumulation

MAMs are crucial in modulating cellular redox status, acting as both regulators of ROS synthesis and targets of oxidative damage (Resende et al., 2022). TEM studies have demonstrated that stimulation with hydrogen peroxide results in a decreased distance between mitochondria and the endoplasmic reticulum (ER), along with an elongation of the MAMs. In contrast, treatment with Rb1 increases the distance between these organelles and reduces the length of the MAMs (Figure 6A). To further explore these interactions, we examined the expression of MAM-associated tether proteins. Our results indicate that Rb1 partially reverses the oxidative stress-induced upregulation of FUNDC1, GRP75, and MFN2 (Figure 6B). We observed that H_2_O_2_ treatment increased the co-localization of the ER protein IP3R with mitochondria, while Rb1 administration reduced the co-localization of IP3R with mitochondria (Figure 6C). IP3R is integral to the mitochondrial calcium channel complex, IP3R/GRP75/VDAC1, which facilitates Ca^2+^ transport (Li et al., 2022). Our findings show that Rb1 treatment significantly reduces mitochondrial Ca^2+^ concentration (Figure 6D). Furthermore, Rb1 mitigates mitochondrial ROS accumulation and enhances MMPs (Figures 6E,F). In summary, Rb1 plays a vital role in maintaining MAM stability under oxidative stress, alleviating calcium overload, reducing mitochondrial ROS accumulation, and thereby contributing to the mitigation of oxidative stress.

**FIGURE 6:**
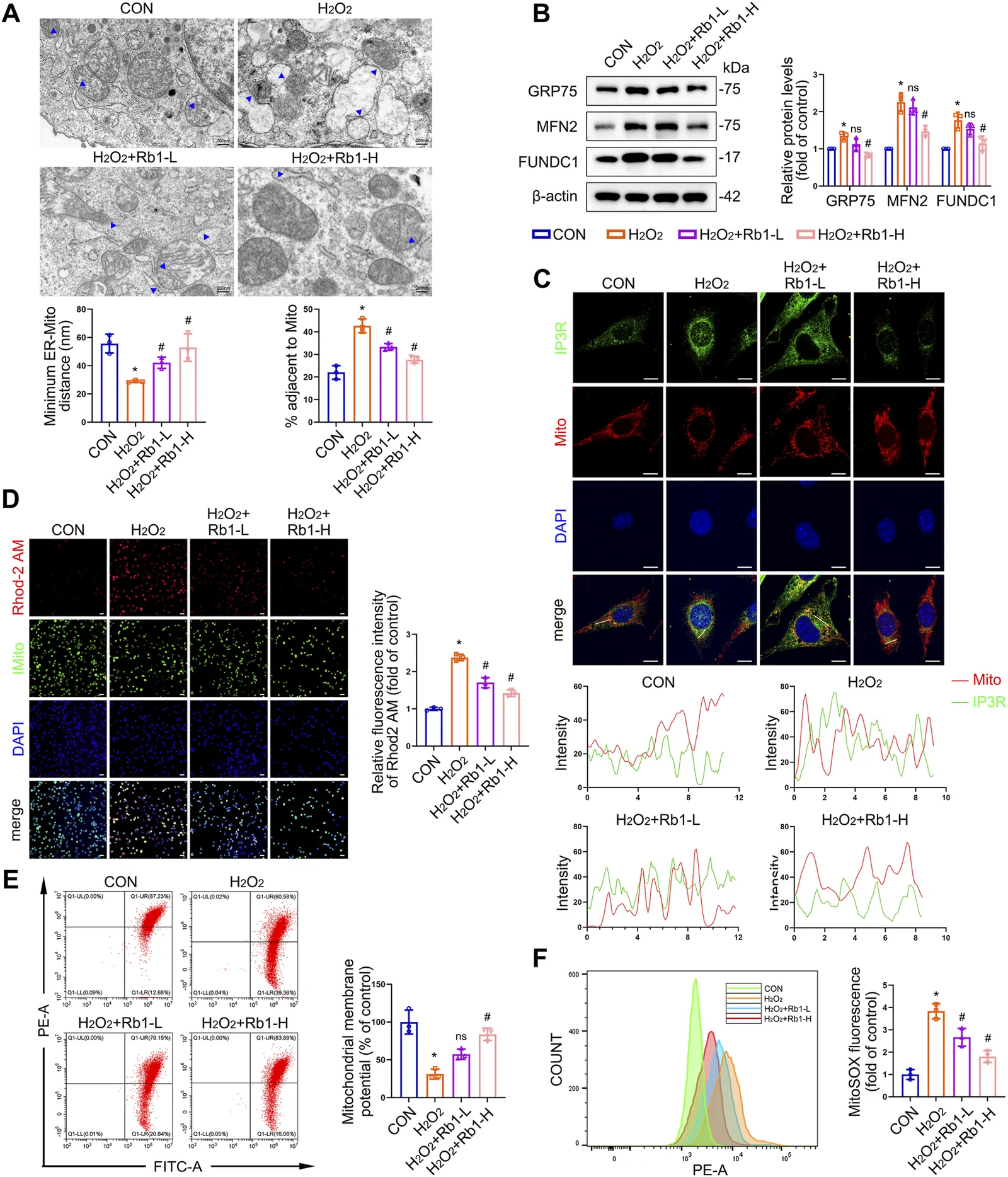
Ginsenoside Rb1 mitigates the damage of MAMs by oxidative stress. **(A)** Representative TEM images of mitochondrial ultrastructure of L929 cells. MAMs were indicated by blue arrows. Quantitative analysis of MAM parameters, including MAM distance and length to mitochondrion in TEM images. **(B)** Immunoblot analyses of GRP75, MFN2 and FUNDC1 in L929 cells. Quantification represents the levels of the indicated protein normalized to β-actin. **(C)** Colocalization analysis of immunofluorescence images of IP3R (green) and Mito-tracker (red) in different groups of L929 cells. Scale bar = 10 μm. **(D)** Immunofluorescence staining of Rhod2 AM (red) and Mito-tracker (green) in L929 cells. Scale bar = 20 μm. **(E)** Representative flow plots of MMP levels in L929 cells measured by flow cytometry. Data are expressed as mean ± SD. **(F)** Representative flow plots of mitochondrial ROS production in L929 cells measured by flow cytometry. Data are expressed as mean ± SD. For all panels, n = 3 independent experiments. *P < 0.05 compared with the control group and ^#^ *P* < 0.05 compared with the H_2_O_2_ group. ns indicates no significant difference.

### 3.6 Ginsenoside Rb1 preserves mitochondrial homeostasis through SIRT1-associated signaling

To explore a potential interaction between ginsenoside Rb1 and SIRT1, we performed molecular docking analysis. The predicted binding energy was −9.17 kcal/mol, suggesting a computationally favorable interaction between Rb1 and SIRT1 (Figure 7A). We further employed siRNA targeting SIRT1 and transfected it into L929 cells. The efficiency of SIRT1 knockdown by siRNA was first validated by Western blotting (Supplementary Figure S1A). SIRT1 expression was upregulated in Rb1-treated L929 cells, and SIRT1 knockdown effectively reduced SIRT1 expression (Figure 7B). SIRT1 knockdown reversed the activation of the PGC-1α/NRF1/TFAM signaling pathway by Rb1 (Figure 7C). Additionally, the promotion of mtDNA content by Rb1 was reversed following SIRT1 knockdown (Figure 7D). The protective effect of Rb1 on mitochondrial morphology was also diminished, as evidenced by an increase in fragmented mitochondria upon SIRT1 knockdown (Figure 7E). Furthermore, the enhancement of PINK1/Parkin-associated signaling by Rb1 was attenuated following SIRT1 knockdown (Figure 7F). The Rb1-induced mitochondrial recruitment of PINK1 and Parkin was similarly reduced by SIRT1 knockdown (Figures 7G,H).

**FIGURE 7:**
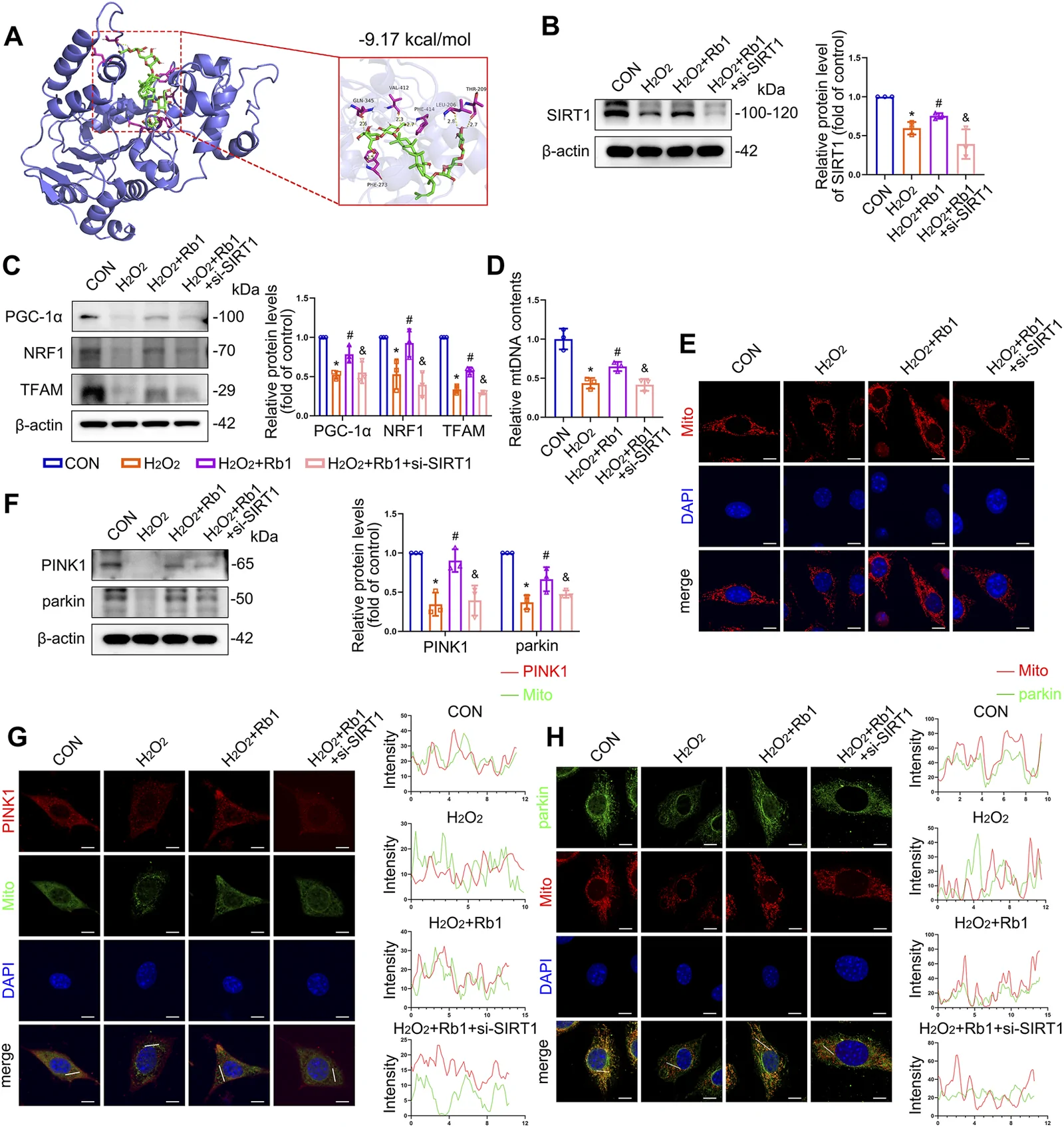
Ginsenoside Rb1 promotes mitochondrial biogenesis-associated signaling and PINK1/Parkin-associated mitophagic signaling through SIRT1. **(A)** Molecular docking of ginsenoside Rb1 with SIRT1. The binding energy is −9.17 kcal/mol. **(B)** Immunoblot analysis of SIRT1 in L929 cells. Quantification represents the level of the indicated protein normalized to β-actin. **(C)** Immunoblot analyses of PGC-1α, NRF1 and TFAM in L929 cells. Quantification represents the levels of the indicated protein normalized to β-actin. **(D)** RT-qPCR analyses of relative mtDNA contents in L929 cells. **(E)** Representative images from Mito-tracker-stained L929 cells. Scale bars = 10 μm. **(F)** Immunoblot analyses of PINK1 and parkin in L929 cells. Quantification represents the levels of the indicated protein normalized to β-actin. **(G,H)** Colocalization analysis of immunofluorescence images of PINK1 (red) and Mito-tracker (green) **(G)**, parkin (green) and Mito-tracker (red) **(H)** in different groups of L929 cells. Data are expressed as mean ± SD. For all panels, n = 3 independent experiments. *P < 0.05 compared with the control group, ^#^ *P* < 0.05 compared with the H_2_O_2_ group, and ^&^ *P* < 0.05 compared with the H_2_O_2_+Rb1 group. ns indicates no significant difference.

To further investigate whether SIRT1 contributes to the protective effects of ginsenoside Rb1 on mitochondrial homeostasis in the anterior vaginal wall of VD mice, we performed an experiment involving intraperitoneal administration of Rb1 combined with the SIRT1 inhibitor EX527 for seven consecutive days following VD (Figure 8A). On the seventh day, we measured the BLPP and assessed histopathological changes in the anterior vaginal wall. Our analyses revealed that while Rb1 treatment increased BLPP levels in VD mice, concomitant EX527 administration attenuated this improvement (Figure 8B). While Rb1 treatment increased BLPP levels in VD mice, EX527 partially inhibited these effects (Figure 8C). The restoration of collagen organization induced by Rb1 was attenuated in VD mice receiving EX527 treatment. (Figures 8D,E). We further investigated whether SIRT1 inhibition affected the beneficial effects of Rb1 on mitochondrial homeostasis. The PGC-1α/NRF1/TFAM axis was inhibited in the anterior vaginal wall of VD mice treated with both Rb1 and EX527, indicating that mitochondrial biogenesis-associated program was suppressed (Figure 9A). Similarly, PINK1/parkin expression was inhibited following EX527 treatment (Figure 9B). These findings suggest that SIRT1 contributes to the effects of Rb1 on mitochondrial homeostasis by regulating mitochondrial biogenesis-associated signaling and PINK1/Parkin-associated mitophagic signaling.

**FIGURE 8:**
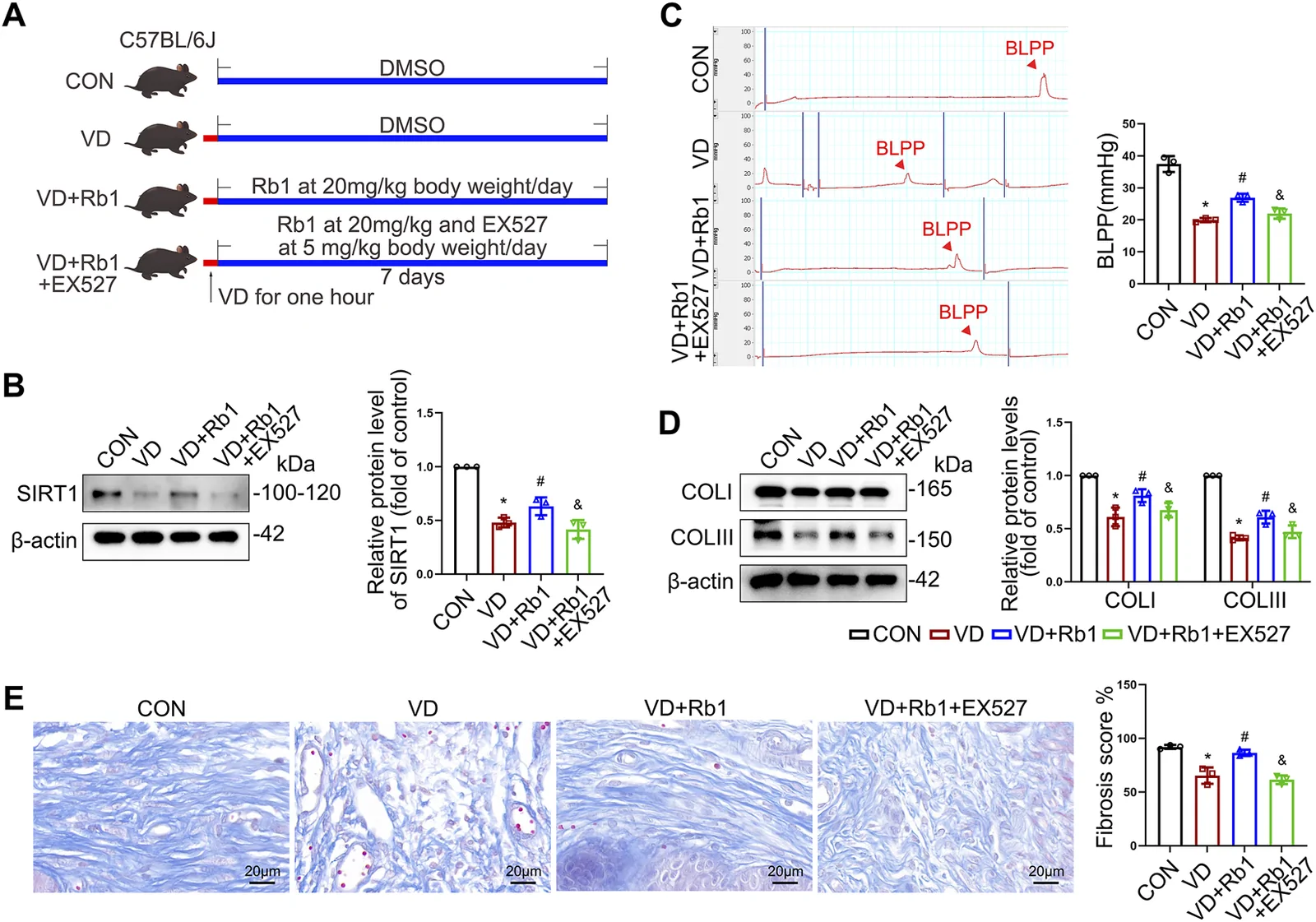
Ginsenoside Rb1 improves pelvic floor support tissue function in SUI mice by upregulating SIRT1. **(A)** Experimental design of VD mice treated with ginsenoside Rb1 and EX527. **(B)** Immunoblot analysis of SIRT1 in anterior vaginal wall in mice. Quantification represents the level of the indicated protein normalized to β-actin. **(C)** The represent-curve of urinary dynamics of different groups, and statistical analysis of BLPP for different groups on 7 days. **(D)** Immunoblot analysis of collagen I (COL I) and collagen III (COL III) in anterior vaginal wall in mice. Quantification represents the level of the indicated protein normalized to β-actin. **(E)** Masson staining for collagen expression in the tissue of the anterior vaginal wall in mice of four groups. Data are expressed as mean ± SD. For all panels, n = 3 mice per group. *P < 0.05 compared with the control group, ^#^ *P* < 0.05 compared with the VD group, and ^&^ *P* < 0.05 compared with the VD + Rb1 group. ns indicates no significant difference.

**FIGURE 9:**
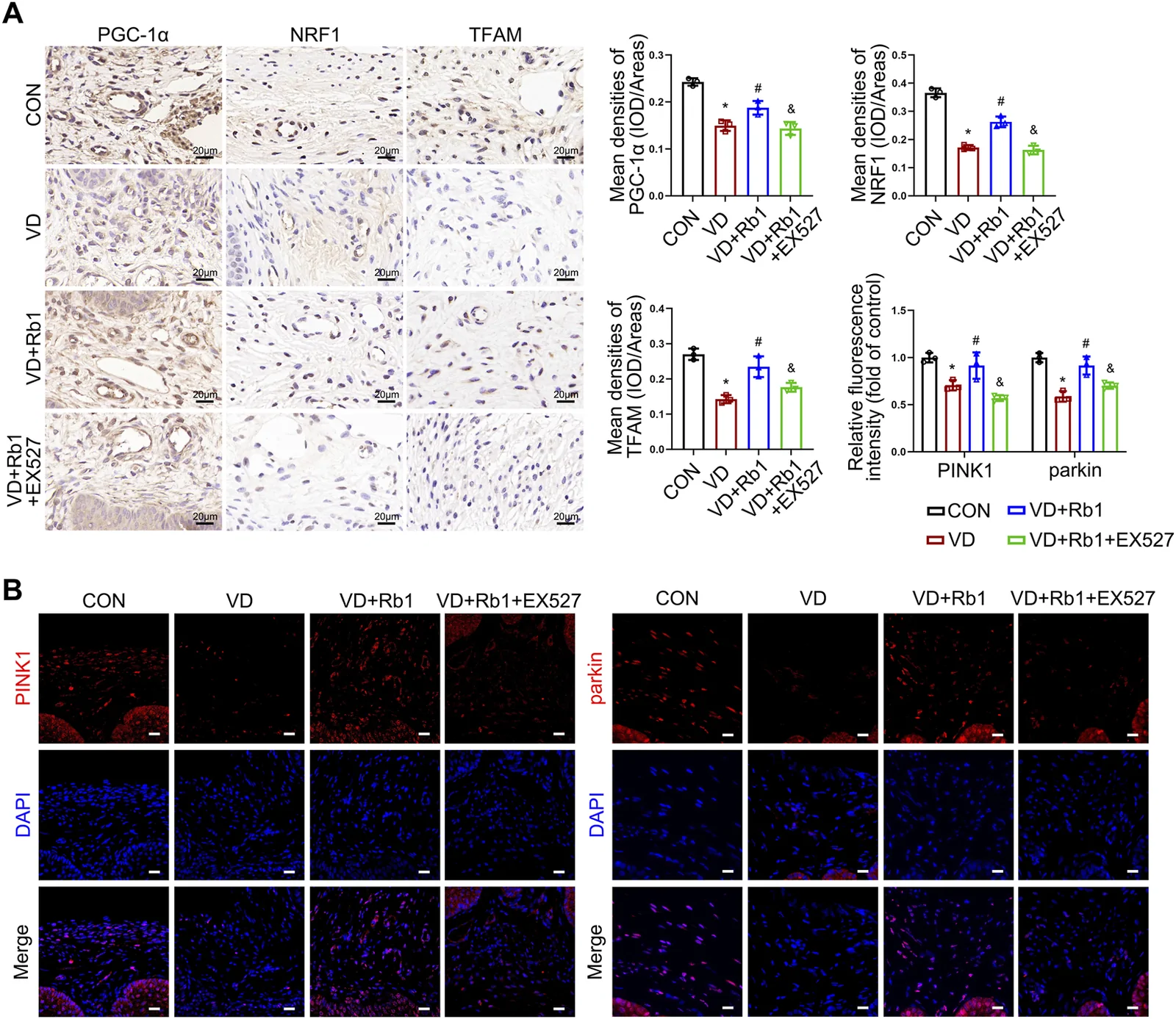
Ginsenoside Rb1 enhances mitochondrial function in fibroblasts by upregulating SIRT1. **(A)** Representative images of the IHC staining for PGC-1α, NRF1 and TFAM in anterior vaginal wall were obtained and quantified. **(B)** Immunofluorescence staining of PINK1 and parkin in the anterior vaginal wall of mice. Scale bar = 20 μm. Data are expressed as mean ± SD. For all panels, n = 3 mice per group. *P < 0.05 compared with the control group, ^#^ *P* < 0.05 compared with the VD group, and ^&^ *P* < 0.05 compared with the VD + Rb1 group. ns indicates no significant difference.

### 3.7 Ginsenoside Rb1 maintains the structural stability of MAMs by promoting the expression of SIRT1

To investigate whether SIRT1 contributes to Rb1-mediated regulation of MAM integrity at the cellular level, we performed SIRT1 knockdown experiments in L929 cells and analyzed MAM structural characteristics using TEM. Our observations revealed that Rb1 restored the distance between mitochondria and the endoplasmic reticulum, as well as the length of the MAM, following oxidative stress. However, this recovery was reversed upon SIRT1 knockdown (Figure 10A). Additionally, the expression levels of MAM tether proteins FUNDC1, MFN2, and GRP75 were significantly elevated after SIRT1 knockdown (Figure 10B). We also noted an increase in the co-localization of IP3R with mitochondria following SIRT1 knockdown (Figure 10C). Furthermore, while Rb1 effectively attenuated mitochondrial calcium overload induced by oxidative stress, its ability to alleviate this overload was compromised by SIRT1 knockdown (Figure 10D). Similarly, the reduction of mtROS accumulation by Rb1 was reversed with SIRT1 knockdown (Figure 10E). To further validate the effects of ginsenoside Rb1 and SIRT1 on MAMs, we conducted TEM analysis on the anterior vaginal wall fibroblasts of VD mice treated with Rb1 and the SIRT1 inhibitor EX527. Our results indicated that EX527 treatment attenuated the protective effects of Rb1 on MAM-associated alterations (Figure 10F). Collectively, these findings suggest that SIRT1 contributes to the ability of Rb1 to preserve MAM integrity and regulate mitochondrial calcium homeostasis and mtROS generation.

**FIGURE 10:**
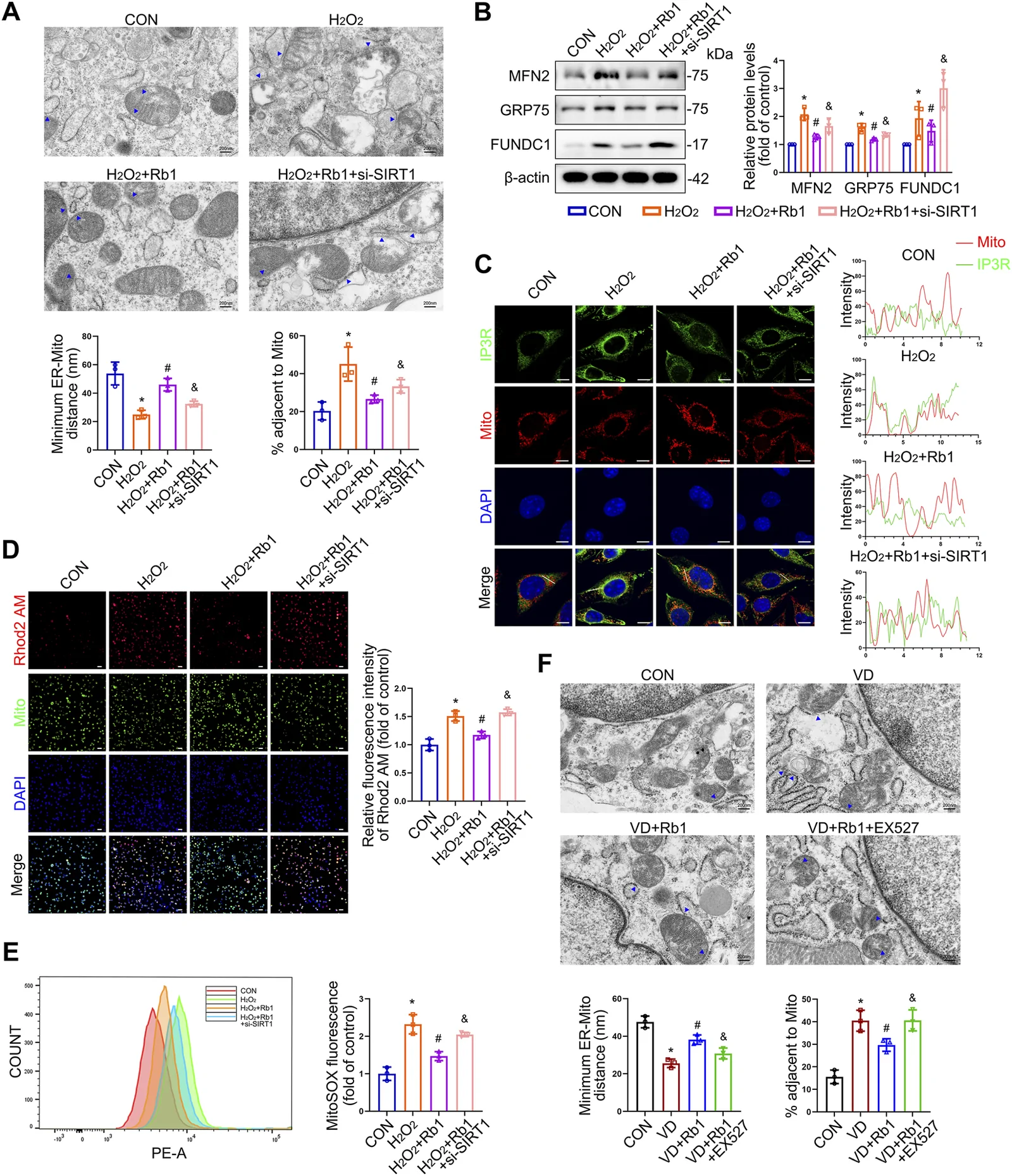
Rb1 preserves MAM structure through SIRT1-mediated inhibition of tether proteins. **(A)** Representative TEM images of mitochondrial ultrastructure of L929 cells. MAMs were indicated by blue arrows. Quantitative analysis of MAM parameters, including MAM distance and length to mitochondrion in TEM images. **(B)** Immunoblot analyses of GRP75, MFN2 and FUNDC1 in L929 cells. Quantification represents the levels of the indicated protein normalized to β-actin. **(C)** Colocalization analysis of immunofluorescence images of IP3R (green) and Mito-tracker (red) in different groups of L929 cells. Scale bar = 10 μm. **(D)** Immunofluorescence staining of Rhod2 AM (red) and Mito-tracker (green) in L929 cells. Scale bar = 20 μm. **(E)** Representative flow plots of mitochondrial ROS production in L929 cells measured by flow cytometry. **(F)** Representative TEM images of mitochondrial ultrastructure of fibroblasts in the anterior vaginal wall of mice. MAMs were indicated by blue arrows. Quantitative analysis of MAM parameters, including MAM distance and length to mitochondrion in TEM images. Data are expressed as mean ± SD. For cell culture experiments **(A–E)**, n = 3 independent experiments. For animal experiments **(F)**, n = 3 mice per group. For **(A–E)**, *P < 0.05 compared with the control group, ^#^ *P* < 0.05 compared with the H_2_O_2_ group, and ^&^ *P* < 0.05 compared with the H_2_O_2_ + Rb1 group. For **(F)**, *P < 0.05 compared with the control group, ^#^ *P* < 0.05 compared with the VD group, and ^&^ *P* < 0.05 compared with the VD + Rb1 group.

### 3.8 Ginsenoside Rb1 preserves MAM homeostasis through the SIRT1-associated regulation of GRP75 acetylation

To further investigate the molecular mechanism associated with SIRT1-dependent regulation of MAM responses in L929 cells, we examined the acetylation status of MAM-associated proteins, including GRP75, MFN2, and FUNDC1. Among these proteins, GRP75 showed the most prominent changes in acetylation. H_2_O_2_ markedly increased GRP75 acetylation, whereas Rb1 treatment reduced this increase, an effect that was largely reversed by SIRT1 knockdown (Figure 11A). Co-immunoprecipitation further confirmed the interaction between SIRT1 and GRP75, suggesting that GRP75 may act as a downstream target of SIRT1 (Figure 11B).

**FIGURE 11:**
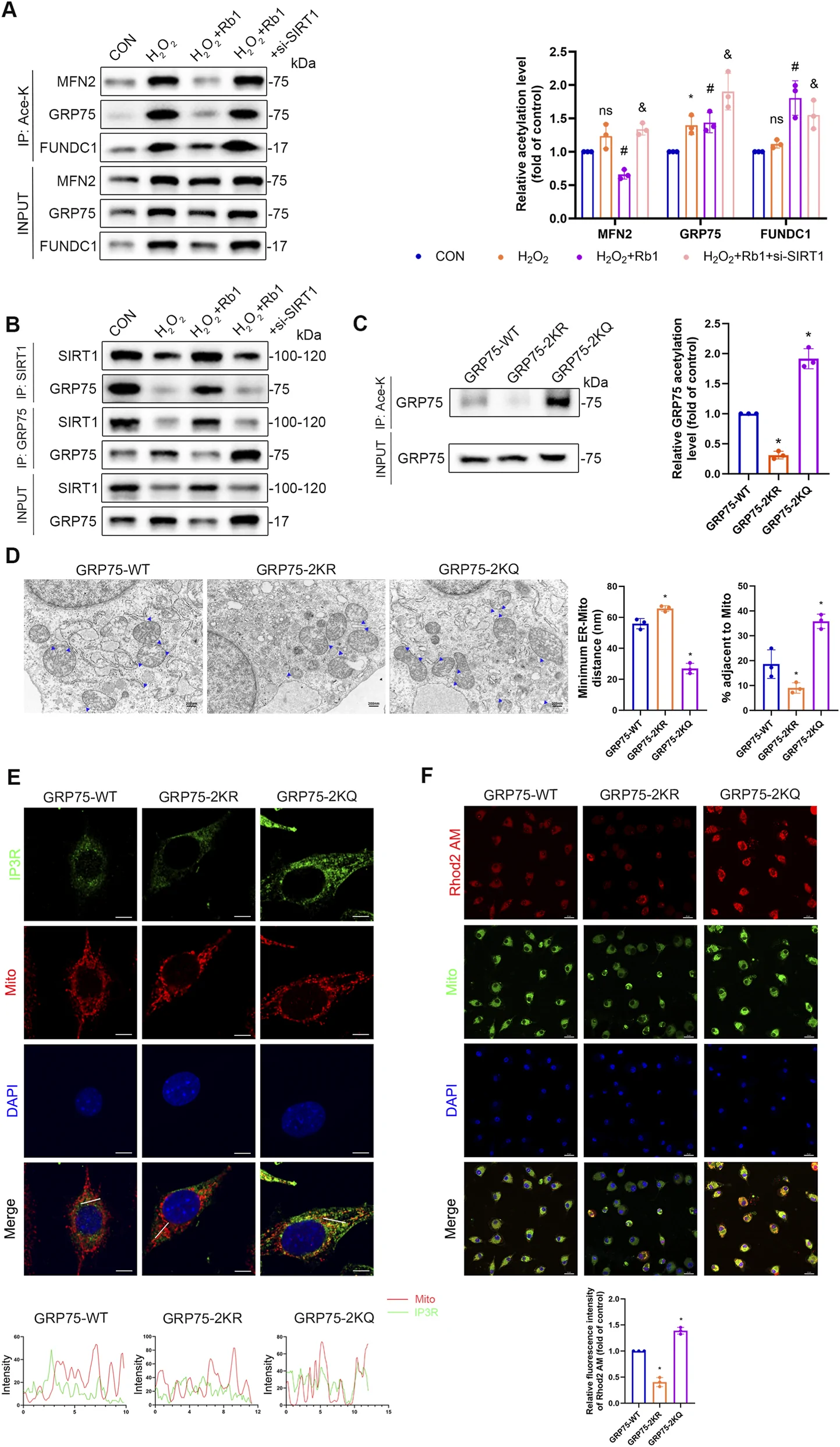
Ginsenoside Rb1 preserves MAM integrity and mitochondrial calcium homeostasis through SIRT1-mediated regulation of GRP75 acetylation. **(A)** Acetyl-lysine-associated signals of GRP75, MFN2, and FUNDC1 were assessed by immunoprecipitation with an anti-acetylated lysine Ac-K antibody followed by immunoblotting. **(B)** Co-immunoprecipitation analysis of the interaction between SIRT1 and GRP75 under the indicated conditions. **(C)** Assessment of the acetyl-lysine-associated signal of GRP75-WT, GRP75-2KR, and GRP75-2KQ by Ac-K immunoprecipitation followed by GRP75 immunoblotting. **(D)** Representative TEM images of mitochondrial ultrastructure of L929 cells. MAMs were indicated by blue arrows. Quantitative analysis of MAM parameters, including MAM distance and length to mitochondrion in TEM images. **(E)** Colocalization analysis of immunofluorescence images of IP3R (green) and Mito-tracker (red) in different groups of L929 cells. Scale bar = 10 μm. **(F)** Immunofluorescence staining of Rhod2 AM (red) and Mito-tracker (green) in L929 cells. Scale bar = 25 μm. Data are expressed as mean ± SD. For all panels, n = 3 independent experiments. For **(A)**, *P < 0.05 compared with the control group, ^#^ *P* < 0.05 compared with the H_2_O_2_ group, and ^&^ *P* < 0.05 compared with the H_2_O_2_+Rb1 group. For **(C–E)**, *P < 0.05 compared with the GRP75-WT group. ns indicates no significant difference.

To investigate the functional role of GRP75 acetylation, GRP75-WT, GRP75-2KR (K567R/K612R, deacetylation-mimetic), and GRP75-2KQ (K567Q/K612Q, acetylation-mimetic) mutants were generated. Ac-K immunoprecipitation was performed to assess the acetyl-lysine-associated signal of these GRP75 constructs (Figure 11C). Compared with GRP75-WT, GRP75-2KR increased the ER–mitochondrial distance and reduced MAM contact length (Figure 11D). Moreover, GRP75-2KR expression significantly reduced the colocalization of IP3R with mitochondria compared with GRP75-WT, indicating impaired ER–mitochondrial interaction and MAM-associated calcium communication (Figure 11E). In parallel, mitochondrial Ca^2+^ levels were significantly decreased in GRP75-2KR-expressing cells, further demonstrating that GRP75 acetylation status influences mitochondrial calcium homeostasis (Figure 11F). Collectively, these findings suggest that Rb1 regulates SIRT1-associated GRP75 acetylation and that the acetylation state of GRP75 is involved in the regulation of MAM integrity and mitochondrial calcium homeostasis.

## 4 Discussion

SUI is a prevalent condition among middle-aged women, predominantly resulting from the weakening of urethral supportive tissues, which substantially affects their quality of life. A principal pathological factor contributing to this tissue weakening is oxidative stress, which disrupts the metabolic equilibrium of the ECM in the anterior vaginal wall, thereby diminishing its supportive capacity for urethral tissues (Kuprasertkul et al., 2021; Sangsawang and Sangsawang, 2013). Prior research has demonstrated that the administration of antioxidants or the overexpression of antioxidant proteins can significantly enhance collagen expression in the anterior vaginal wall, consequently improving urodynamic function in SUI mouse models (Li et al., 2021b; Lu et al., 2023; Zhang et al., 2017). This underscores the critical importance of addressing oxidative stress in the anterior vaginal wall of SUI patients. In our study, we observed that ginsenoside Rb1 can mitigate oxidative stress and restore collagen metabolism in the anterior vaginal wall of VD mice, resulting in improved urodynamic outcomes.

Fibroblasts serve as the principal resident cells responsible for the synthesis, remodeling, and mechanical properties of the ECM within pelvic floor connective tissues. In comparison to other cell types, fibroblasts exhibit elevated metabolic activity, necessitated by their continuous demands for protein synthesis, collagen production, and matrix turnover (Babaei et al., 2016; Guler and Roovers, 2022). These processes require a substantial supply of mitochondrial energy and the maintenance of tightly regulated redox homeostasis. Consequently, mitochondrial dysfunction in fibroblasts may directly compromise ECM remodeling by diminishing cellular energy production, increasing oxidative damage, and promoting apoptosis or phenotypic changes associated with senescence (Schuliga et al., 2018; Song et al., 2023). Under pathological mechanical stress, such as that induced by vaginal distension, excessive accumulation of ROS may disrupt mitochondrial quality control mechanisms in fibroblasts, leading to impaired collagen synthesis and accelerated degeneration of connective tissue (Liu et al., 2025). Therefore, the preservation of mitochondrial function in fibroblasts is a critical mechanism for maintaining the integrity of pelvic floor tissues.

Ginsenoside Rb1 is recognized as a potent natural antioxidant, notable for its capacity to scavenge free oxygen radicals (Ahmed et al., 2016). It alleviates oxidative stress by promoting the ubiquitin-mediated degradation of Keap1, thereby facilitating the nuclear accumulation and activation of Nrf2. This process induces the nuclear translocation of Nrf2, which subsequently upregulates the expression of the antioxidant enzyme HO-1 (Chen et al., 2023; Wang et al., 2022). Mitochondria, which are particularly vulnerable to oxidative damage, benefit from Rb1’s ability to restore their structure and function through the enhancement of mitochondrial biogenesis (Dong et al., 2022). Recent investigations have further elucidated the mechanisms underlying Rb1-mediated mitochondrial protection. Rb1 has been demonstrated to preserve functional mitochondria and facilitate mitochondrial transfer, thereby safeguarding cells against oxidative injury. Additionally, formulations containing Rb1 have been reported to restore mitochondrial ultrastructure by increasing mitochondrial abundance and alleviating mitochondrial swelling, thereby reinforcing the role of Rb1 in maintaining mitochondrial integrity and homeostasis (He et al., 2025; Ni et al., 2022). Furthermore, Rb1 reduces the generation of reactive oxygen species by stimulating mitophagy (Zhang et al., 2023; Zhang R. et al., 2024). Nonetheless, research on the impact of Rb1 on enhancing mitochondrial function remains limited.

In this study, L929 cells were selected as the *in vitro* model because fibroblasts are the main stromal cells involved in the synthesis and remodeling of extracellular matrix in pelvic floor connective tissue (Zhang and Zhu, 2026). L929 cells are extensively utilized as a reliable fibroblast model because of their stable phenotype, consistent responses to oxidative stress, and amenability to molecular manipulation. Although primary vaginal fibroblasts may more accurately represent the physiological characteristics of human pelvic tissues, their limited availability, variability between donors, and challenges associated with genetic manipulation constrain their use in mechanistic studies. Consequently, L929 cells were used as a controlled cellular model to dissect the molecular mechanisms associated with oxidative stress-induced mitochondrial dysfunction, whereas VD mouse models were used to evaluate the physiological effects of Rb1 on anterior vaginal wall integrity and urodynamic function.

SIRT1 exhibits a high sensitivity to alterations in the cellular redox state (Winnik et al., 2015; Zheng et al., 2020) and plays a crucial role in enhancing pelvic floor support by mitigating oxidative stress and cellular senescence in human uterosacral ligament fibroblasts (Wang et al., 2024). Previous research has demonstrated that various ginsenoside monomers confer therapeutic benefits across a range of diseases through the modulation of SIRT1 (Huang et al., 2021; Yin et al., 2024). In light of these findings, we conducted an investigation into the effects of Rb1 treatment on SIRT1 expression both *in vitro* and *in vivo*. Our results demonstrated that Rb1 restored SIRT1 expression under oxidative stress conditions. SIRT1 inhibition or knockdown attenuated the activation of PGC-1α/NRF1/TFAM-associated mitochondrial biogenesis signaling and PINK1/Parkin-associated mitochondrial quality-control signaling induced by Rb1, thereby weakening its protective effects on collagen metabolism and urodynamic function. These findings suggest that SIRT1 is an important contributor to Rb1-mediated mitochondrial homeostasis in fibroblast models and may contribute to the protective effects of Rb1 against SUI.

In recent years, the interactions between organelles have attracted significant scholarly attention (Xu et al., 2020a; Xu et al., 2020b). Researchers have identified a direct physical connection, known as the MAM, between mitochondria and the endoplasmic reticulum, which plays a critical role in facilitating inter-organelle communication (Vance, 1990; Wieckowski et al., 2009). Oxidative stress is closely associated with mitochondrial calcium overload, and MAMs function as a conduit for Ca2+ transport between mitochondria and the endoplasmic reticulum (Li et al., 2024; Luo et al., 2024; Zhang Z. et al., 2024). Our investigations demonstrated that oxidative stress decreased the distance between mitochondria and the endoplasmic reticulum, increased the length of MAMs, and exacerbated calcium overload. However, treatment with Rb1 significantly ameliorated these alterations in MAMs and reduced both mitochondrial Ca2+ levels and mtROS levels.

MAM remodeling may contribute to mitochondrial dysfunction and tissue homeostasis in SUI. MAMs function as dynamic signaling platforms that orchestrate mitochondrial metabolism, lipid exchange, autophagy initiation, and cellular stress responses (Bui et al., 2026). In fibroblasts, these processes are intricately linked to the maintenance of ECM homeostasis. Excessive or prolonged coupling between the endoplasmic reticulum and mitochondria can result in mitochondrial calcium overload, which subsequently leads to increased production of mitochondrial reactive oxygen species mtROS and the activation of oxidative stress pathways. This oxidative milieu can impair collagen synthesis, stimulate the activation of matrix metalloproteinases, and accelerate ECM degradation, ultimately compromising the mechanical support function of the anterior vaginal wall (Arruda et al., 2014; Zhang et al., 2021). Consequently, restoration of MAM integrity may represent a potential mechanism through which mitochondrial function and connective tissue homeostasis are regulated during SUI progression.

Numerous proteins are involved in the regulation of MAMs. Among these, GRP75 plays a crucial role by facilitating Ca^2+^ transfer between mitochondria and ER through its interaction with IP3R and VDAC1, forming the VDAC1-GRP75-IP3R complex (Mao et al., 2022; Wu et al., 2017). Additionally, FUNDC1, a protein located on the outer mitochondrial membrane, contributes to MAM formation and interacts with IP3R to enhance Ca^2+^ transport from the ER to mitochondria (Wang et al., 2021; Wu et al., 2016). In our study, we investigated the expression levels of these MAM-associated proteins and observed that oxidative stress led to an upregulation of GRP75, MFN2, and FUNDC1. Conversely, treatment with Rb1 resulted in a reduction of their expression, indicating Rb1’s potential role in modulating MAM dynamics. Notably, the expression of these proteins was further elevated upon SIRT1 knockdown. Both *in vivo* and *ex vivo* experiments demonstrated that diminished SIRT1 expression compromised the protective effects of Rb1 on MAMs.

Protein acetylation is an important post-translational modification that regulates diverse cellular processes, including mitochondrial metabolism, protein stability, and organelle communication. Although SIRT1 has been widely recognized as a NAD^+^-dependent deacetylase that regulates mitochondrial function mainly through transcriptional regulators such as PGC-1α, accumulating evidence suggests that SIRT1 also directly modulates mitochondrial proteins through deacetylation, thereby influencing mitochondrial dynamics, calcium homeostasis, and stress responses (Sun et al., 2022; Wang et al., 2020). Therefore, SIRT1-mediated protein deacetylation may represent an additional mechanism linking metabolic stress signals with mitochondrial quality control. In this context, our mechanistic investigation identified GRP75 as a potential downstream effector of SIRT1-mediated MAM regulation. SIRT1 deficiency increased GRP75 acetylation, whereas Rb1 treatment reduced this modification. Functional assays using GRP75 acetylation-mimetic mutants further suggested that GRP75 acetylation status contributes to the regulation of ER–mitochondrial contact characteristics and mitochondrial calcium handling.

Nonetheless, several limitations of this study should be acknowledged. First, although L929 cells provided a useful and experimentally tractable model for investigating oxidative stress-related mitochondrial mechanisms, they cannot fully recapitulate the characteristics of primary human pelvic floor fibroblasts or the complex pathological responses induced by vaginal distension. The H_2_O_2_-induced oxidative stress model represents a reductionist approach that primarily reflects redox-dependent cellular responses rather than the full spectrum of VD-associated tissue injury. Therefore, further validation using primary patient-derived fibroblasts and more physiologically relevant models will be necessary. Second, although SIRT1 knockdown and pharmacological inhibition support an important role for SIRT1 in the protective effects of Rb1, conditional SIRT1 knockout models would provide more definitive *in vivo* evidence for its causal involvement. In addition, the absence of a VD + EX527-only group precludes assessment of the independent effect of EX527 in the VD-induced SUI model. Third, although our findings suggest that Rb1 modulates SIRT1-associated signaling, the precise molecular interaction between Rb1 and SIRT1 remains to be elucidated. Furthermore, the relatively small number of animals used in the *in vivo* experiments (n = 3 per group) and the absence of a formal *a priori* power calculation may limit the statistical power and generalizability of the mechanistic findings. Finally, further studies are needed to evaluate the long-term efficacy, safety, and clinical applicability of Rb1 for the treatment of SUI.

## 5 Conclusion

In conclusion, this study elucidates that ginsenoside Rb1 confers protection against stress urinary incontinence by reestablishing mitochondrial homeostasis through modulation of mitochondrial homeostasis in cellular and tissue models. Mechanistically, our findings suggest that Rb1 acts through SIRT1-associated signaling to enhance mitochondrial biogenesis-associated responses via the PGC-1α/NRF1/TFAM pathway and to enhance PINK1/Parkin-associated mitochondrial quality-control signaling. Furthermore, SIRT1-associated regulation of GRP75 acetylation maintains the integrity of MAMs, thereby mitigating mitochondrial calcium overload and excessive mtROS accumulation. By orchestrating the regulation of mitochondrial quality control and MAM-dependent calcium homeostasis, Rb1 mitigates oxidative stress, sustains collagen metabolism, and enhances the function of the anterior vaginal wall. These findings identify the SIRT1–GRP75/MAM axis as a potential mechanism contributing to the protective effects of Rb1 and suggest a potential therapeutic approach for addressing stress urinary incontinence.

## Data Availability

The raw data supporting the conclusions of this article will be made available by the authors upon reasonable request.

## Glossary

SUI: stress urinary incontinence
POP: pelvic organ prolapse
SIRT1: Sirtuin 1
Rb1: ginsenoside Rb1
VD: vaginal dilatation
RT-qPCR: reverse transcription-quantitative real-time PCR
MAMs: mitochondria-associated membranes
TEM: transmission electron microscopy
siRNA: small interfering RNA
mitoROS: mitochondrial reactive oxygen species
MMP: mitochondrial membrane potential
BLPP: bladder leak point pressure
PGC-1α: peroxisome proliferator-activated receptor gamma, coactivator 1 alpha
NRF1: nuclear Respiratory Factor 1
TFAM: mitochondrial transcription factor 1
PINK1: PTEN-induced putative kinase protein 1
NAD: nicotinamide adenine dinucleotide
H2O2: hydrogen peroxide
ROS: reactive oxygen species
Nrf2: nuclear factor erythroid 2-related factor 2
LC3B: microtubule associated protein 1 light chain 3 beta
BAX: BCL2-associated X protein
Bcl2: B-cell lymphoma 2
mtDNA: mitochondrial DNA
8-OHdG: 8-hydroxy-2′-deoxyguanosine
4-HNE: 4-hydroxynonenal
TUNEL: terminal deoxynucleotidyl transferase mediated dUTP nick end labeling
IP3R: inositol 1,4,5-triphate receptor
VDAC1: voltage-dependent anion channel-1
GRP75: glucose regulated protein 75
FUNDC1: FUN14 domain containing 1
