# Supplementary material for: Ginsenoside Rb1 ameliorates stress urinary incontinence by upregulating SIRT1 to preserve mitochondrial homeostasis and MAM integrity
Source: Front Pharmacol. 2026 Oct 2;17:1912811. doi: 10.3389/fphar.2026.1912811 (PMC13672532; doi:10.3389/fphar.2026.1912811)
Supplement: Supplementary file 1 [file Table1.docx]

**Ginsenoside Rb1 ameliorates stress urinary incontinence by upregulating SIRT1 to preserve mitochondrial homeostasis and MAM integrity**

***Supplementary materials***

**Supplementary** **tables**

**Table S1. Sequences of primers used in RT-qPCR for measurement of relative mtDNA contents**

| Gene | Species | Forward primer | Reverse primer |
| --- | --- | --- | --- |
| mtND4 | HUMAN | CTCATCAGTAAGCCATATAGC | TTCGTTCGTAGTTGGTGTT |
| mtGAPDH | HUMAN | GGAAGGACTCATGACCACAGT | GCCATCACGCCACAGTTTC |
| mtAtp6 | MICE | GCCATTCCACTATGAGCTGGAGCC | GTGGAAGGAAGTGGGCAAGTGAGC |
| Tert | MICE | CTAGCTCATGTGTCAAGACCCTCTT | GCCAGCACGTTTCTCTCGTT |

**Supplementary** **figures**

**Figure S1. Validation of SIRT1 knockdown efficiency and Rb1-induced activation of the PGC-1α/NRF1/TFAM signaling axis. (A)** Western blot analysis of SIRT1 expression in L929 cells transfected with three independent SIRT1-targeting siRNAs or si-NC. Quantification represents the level of SIRT1 protein normalized to β-actin. **(B–D)** Immunofluorescence staining of PGC-1α (B), NRF1 (C), and TFAM (D) in L929 cells. Scale bars = 20 μm. Data are expressed as mean ± SD. For all panels, n = 3 independent biological experiments. In panel (A), *P < 0.05 compared with the si-NC group. In panels (B–D), *P < 0.05 compared with the control group, and #P < 0.05 compared with the H₂O₂ group. ns indicates no significant difference.
